# Disaster Preparedness Training for Emergency Medicine Residents Using a Tabletop Exercise

**DOI:** 10.15766/mep_2374-8265.11119

**Published:** 2021-03-12

**Authors:** Ariel Sena, Frank Forde, Catherine Yu, Harsh Sule, M. Meredith Masters

**Affiliations:** 1 Assistant Professor of Emergency Medicine, Department of Emergency Medicine, Rutgers New Jersey Medical School; 2 Clinical Instructor of Emergency Medicine and Assistant Director of Prehospital and Disaster Medicine, Department of Emergency Medicine, University Hospitals Cleveland Medical Center; 3 Resident, Department of Emergency Medicine, Rutgers New Jersey Medical School; 4 Associate Professor of Emergency Medicine, Residency Program Director, and Associate Director, Rutgers New Jersey Medical School Office of Global Health, Rutgers New Jersey Medical School; 5 Assistant Professor, Department of Emergency Medicine, Rutgers New Jersey Medical School; Medical Director, Emergency Medical Services, University Hospital

**Keywords:** Emergency Medicine, Disaster Medicine, Tabletop Exercise, Games

## Abstract

**Introduction:**

Emergency medicine (EM) physicians serve at the frontline of disasters in our communities. The 2016 Model of Clinical Practice according to the American Board of EM identifies disaster management as an integral task of EM physicians. We described a low-cost and feasible tabletop exercise to implement such training for EM residents.

**Methods:**

The exercise took place during 2 hours of resident didactic time. A lecture introduced the incident command system (ICS) and triage concepts, followed by a tabletop scenario with a map of a disaster scene or emergency department. Facilitators presented situational prompts of tasks for residents to address during the exercise. These exposed residents to challenges in disaster scenarios, such as surge and limited resources. The exercise concluded with a debrief and short lecture reviewing scenario-specific topics and challenges. Residents completed an online pre- and postexercise assessment, evaluating knowledge and perceptions of disaster scenario management.

**Results:**

Eighteen residents participated in this exercise. The response rates to the pre- and postsurvey were 76% and 72% respectively. Using a Mann Whitney U test, no statistically significant difference was demonstrated on the medical knowledge component of the survey. There was, however, a statistically significant increase in perceived confidence of the residents' ability to manage disaster incidents.

**Discussion:**

We developed a simple exercise that is an easily adaptable and practical option for introduction to disaster preparedness training. These concepts are difficult to teach and assess among learners, however it remains an important component of education for EM physicians-in-training.

## Educational Objectives

By the end of this activity, learners will be able to:

1. Apply knowledge of concepts of a mass casualty incident and disaster preparedness such as incident command system (ICS), simple triage and rapid treatment, and surge capacity management in a simulated disaster experience.
2. Collaborate with colleagues to quickly and efficiently form a team, and identify roles as described in the ICS.
3. Demonstrate the ability to communicate with team members effectively.
4. Describe the ethical dilemmas associated with disaster medicine, such as staff utilization and resource allocation, and describe the ways to anticipate and manage some of these dilemmas.

## Introduction

The definition of disaster has been disputed, but is recognized by the United Nations Disaster Management Training Program as, “A serious disruption of the functioning of a society, causing widespread human, material or environmental losses which exceed the ability of the affected society to cope using only its own resources.”^1^ The number of casualties needed to be defined as a disaster is not identified, as it depends on the resources available to the affected society.^1^ Both manmade and natural disasters however can quickly overwhelm a system.^1^ From environmental disasters like Hurricane Harvey to mass shootings and more recently the COVID-19 pandemic, disasters are occurring more frequently.^2^ The number of mass shooting incidents in the USA has increased over the last 10 years.^3^ Regardless of the type or location of a disaster, emergency medicine (EM) physicians must be ready at all times for the arrival of large numbers of patients. Such a surge could overwhelm our hospital capacities and introduce patients who are potentially contaminated with chemicals, biological agents, or radioactive material. In the 2016 Model of the Clinical Practice of EM, the American Board of EM (ABEM) identified the importance of the EM physician's ability to practice mass casualty and disaster management.^4^

Despite this, health care facilities remain inadequately prepared for disaster situations.^5^ The Hospital Preparedness Program is a national program implemented by the federal government to improve hospital preparedness for disasters.^6^ While the importance of disaster preparedness remains a high priority at the national or federal level, local hospitals, which are vital to a proper disaster response, have traditionally experienced disasters much more rarely and have therefore made disaster preparedness of lower priority.^2^ Validated methods of assessing hospital preparedness for disasters are lacking, and thus much of what we suspect about preparedness comes from expert opinion.^5–6^ The recent COVID-19 pandemic, however, quickly brought to attention the importance of appropriate disaster planning and surge capacity at widespread local levels.^7^ Physicians could be better equipped to contribute to appropriate planning and preparedness with more training and education around these concepts during residency. According to recent surveys, residents do not feel they receive appropriate training in disaster medicine.^8^ Emergency medical services (EMS) education, which includes disaster preparedness, is recognized by the ACGME as a required component of EM training and as a core component of systems-based practice.^9^ EM physicians are expected to master the ability of task switching and efficiently manage the flow of an emergency department (ED) under high volume or surge situations.^9^ A survey revealed that adding disaster preparedness training to curricula is highly desired by residency directors.^10^

There is a lack of data about which strategies are best to implement this education; however, practical hands-on experience is thought to provide individuals with the most efficient and valid learning opportunities.^10^ Although they are becoming more common, the overall rarity of disasters does not allow most residents to gain firsthand experience.^5^

Exercises are frequently used in disaster planning to monitor and evaluate parts of emergency preparedness training.^11^ A tabletop exercise is a specific type of exercise where a scenario is presented to a group involved in the disaster response.^11^ As the scenario unfolds, the parties involved must explain what actions should be taken and explain how these actions would be implemented.^11^ We saw this format as a potential opportunity to introduce concepts of disaster medicine and challenges experienced during real-life disasters to EM residents in training.

Prior disaster medicine training and exercises exist, however these are frequently large-scale simulation exercises with standardized patients. While this type of simulation has many benefits to learners, conducting these exercises is both costly and time consuming.^12–14^ Such activities are undoubtedly difficult for smaller departments and residency programs to accomplish. To our knowledge, no low-fidelity module for disaster preparedness exists.

We have developed a tabletop exercise to help address this gap in disaster preparedness education among EM residents. It is low cost and requires little preparation, allowing for easy adaptability for various programs, departments, and disaster scenarios.

## Methods

This curriculum was designed for PGY 1–4 level learners in the Rutgers New Jersey Medical School EM residency. It took place during a 2-hour period of the scheduled didactic conference time. This tabletop exercise has been conducted for this group of residents a total of three times over 2 years (2017–2018). No prior knowledge was required by the learners. Facilitators of the exercise consisted of faculty and residents with a special interest in disaster medicine. One of the authors (M. Meredith Masters) was the current EMS medical director who had completed a fellowship in EMS and disaster medicine.

The exercise began with a lecture (Appendix A), about 15–20 minutes in length, introducing learners to concepts of the incident command system (ICS) and simple triage and rapid treatment (START). Residents were divided into two groups of eight to 12 individuals for participation in the tabletop exercise. More or fewer groups can be formed based on the number of learners. This exercise was conducted over the course of 1 hour. Groups encountered the same scenario and worked through it simultaneously. After completion of the tabletop exercise, the groups came back together for a debrief, followed by a postexercise lecture about 30 minutes in length.

For the tabletop exercise, each group had one facilitator who presented the group with situational prompts and encouraged the residents to develop solutions to the problems. We provided all facilitators with detailed instructions on how to guide participant groups through the scenario using timed delivery of patients, feedback on utilization of resources, and other specific prompts (Appendices B and C). The instructions also described how to give or take away supplies with the goal of simulating real strains on resources.

When we conducted this exercise in May 2018, both groups of learners experienced the same disaster scenario. During other presentations of this exercise, we made changes to the exercise to provide the residents with a slightly different experience, while allowing them to continue the practice of disaster preparedness. We provided examples of two of the scenarios we created for a curriculum of this nature experienced by our residents over the multiple presentations of this drill.

### Disaster Scene Experience (Appendix B)

#### Preparation

A general map of a local sports arena was found on the internet and printed on large poster paper. This can be repeated for different local sites with potentials for disasters. A generic map can also be used. It is important to include space for participants to indicate areas for staging, entry, exit, and other operations of the disaster response. A list of nearby hospitals, their distances, and trauma level certifications were written on the map. This list of hospitals can also be adjusted to mirror the participants' local area. Pieces of colored paper or sticky notes were provided for participants to choose areas on the map indicating triage and treatment areas. Mock patients and supplies were printed and placed on index cards. Additional descriptions of the patients on reassessment were also printed on index cards. These patient index cards were then placed in specific zones according to the facilitator guide.

#### Presentation of exercise

The group received the map and a description of the disaster that took place. Facilitators presented different situational prompts with tasks for the participants to complete, including, but not limited to: establishing triage and treatment areas, assigning roles amongst themselves based on ICS, triaging a mass influx of patients, and distributing patients to surrounding hospitals. Facilitators timed the delivery of these prompts according to a provided timeline. Participants were also provided a limited number of resources including endotracheal tubes, ventilators, paramedics, and ambulances, and were faced with challenges on how to allocate them appropriately. The scenario concluded when the group triaged, treated, and transported all patients to nearby hospitals accordingly.

### Hospital Scene Experience (Appendix C)

#### Preparation

A map of our ED was drawn on poster paper with moveable curtains indicated by sticky notes. Descriptions of patients were written on index cards. Some of these patient descriptions represented patients regularly encountered in our ED. These were placed ahead of time on the map in what were considered to be appropriate beds. A list was made with each of these patients' descriptions simulating a sign-out list. Facilitators received a similar list, which contained more details on developments in the courses of the listed patients. The remaining patient descriptions on index cards represented patients that were to present to the ED later in the scenario. These included both mass casualty victims and others with chief complaints unrelated to the disaster. These index cards were held by the facilitator until prompted to distribute to the group according to the facilitator guide and provided timeline. A collection of index cards was given to the group representing supplies available to them including a finite number of available endotracheal tubes, ventilators, and other essential equipment.

#### Presentation of exercise

On arrival, participants were given the sign-out list with the description of patients already present in the ED. Similar to the disaster scene experience, participants were then told of a nearby disaster scenario that took place. Facilitators referred to the guides to prompt the groups to complete specific tasks. These situational prompts were spaced out according to the prepared timeline and completion of tasks to simulate the evolving challenges faced in a disaster. These tasks included establishing an ICS, assigning roles amongst themselves, and designating areas of the ED for triage and treatment. The tasks also included activating on-call staff, addressing the need for additional beds, and assessing the security and safety of the department. The exercise was completed when all mass casualty incident victims were appropriately triaged and treated.

### Exercise Debrief and Concepts Lecture

The tabletop scenario was completed in the course of 1 hour. The residents from all groups came together at the conclusion for a debriefing session. They were encouraged to reflect on what went well as a team, the demands and surprises of the exercise, how they could improve, and how this applied to real-life challenges we may see in our ED. If desired, the facilitator may incorporate a more structured debrief method, such as the plus/delta model, where participants are encouraged to self-reflect in order to identify things that went well and areas of improvement.^15^

The debrief was followed by a 20–30-minute lecture reviewing specific teaching points related to the exercise including surge capacity and blast injuries. The topics changed each time the exercise was conducted to reflect the specific knowledge content encountered in the disaster scenario presented.

### Surveys of the Participants

In May 2018, residents took a survey before and after the exercise (Appendices D and E). This was the second exercise of this type experienced by the residents. Both groups for this particular exercise encountered only the disaster scene scenario. The first part of these surveys included multiple-choice questions testing the residents' knowledge of disaster medicine and EMS concepts. The second part asked questions about their perceptions of the exercise including its effectiveness at preparing them for a real-life disaster scenario. These responses were measured on a 5-point Likert scale. The presurvey was sent electronically to all 29 residents and all responses were anonymous. The postsurvey was sent to the residents who were present for the exercise after its completion. It was impossible to predict exactly who would be present for the tabletop exercise during the protected didactic time and was therefore the reason for initially sending the presurvey to all residents. Completion of both surveys was on a volunteer basis. There was no compensation for completion of the surveys. Residents completed informed consent waivers before taking the surveys. This study was approved by the Rutgers Institutional Review Board.

#### Medical knowledge

In the pre-exercise survey, residents were given eight multiple-choice questions assessing their knowledge of EMS and START concepts. These were written by the authors to reflect concepts that are recognized by the ABEM as core concepts for both the EM in-training examination and the qualifying examination required for board certification. The questions on the postexercise survey tested the same topics but were different questions.

#### Confidence perceptions

For the perceptions component of the pre- and postexercise survey, residents were presented with a hypothetical mass casualty scenario. Prior to the exercise, residents were asked, “Do you feel that your education and training in residency has prepared you adequately to handle this type of incident?” Residents were asked both pre- and postexercise about their confidence in their ability to handle an incident similar to the mass casualty incident described. On the postexercise survey, participants were asked if they believed that a tabletop exercise was an effective education tool for disaster preparedness and training.

#### Analysis

The medical knowledge portions of the pre- and postexercise surveys were analyzed separately from the questions assessing residents' perceptions. The Wilcoxon Rank Sum test was used to determine if the results of both the medical knowledge and perceptions sections were normally distributed. The Mann Whitney U test was used to determine statistical significance.

## Results

A total of 18 out of 29 residents participated in the tabletop exercise. The breakdown by EM year (EMY) is as follows: three in EMY 4, four in EMY 3, four in EMY 2, and seven in EMY 1. There was a 76% (22 of 29) response rate to the pre-exercise survey and a 72% response rate (13 of 18) to the postexercise survey. There was no statistically significant increase in scores on the medical knowledge component of the surveys (63% to 64%, *p* = .873).

All 22 residents who completed the pre-exercise survey reported feeling unprepared. From pre- to postsurvey there was no statistically significant difference in the perception of the importance of disaster medicine training in residents (5 to 4.5 out of 5, *p* = .704). There was a statistically significant increase in residents' self-reported confidence from 2 at presurvey to 4 at postsurvey on a 5-point Likert scale (*p* = .011). On the postexercise survey, all residents agreed that a tabletop exercise was an effective education tool.

## Discussion

The ability to practice disaster medicine and manage mass casualty incidents is recognized by the ABEM as an important skill of EM physicians,^4^ however a gap exists in this training in EM residencies.^8^ Data from our study further supported this notion. While disaster preparedness is something that residency directors desire to add to current curricula,^10^ previous traditional methods for teaching mass casualty and disaster management have been difficult to implement, especially for smaller programs with limited funds and resources.^12–14^ Though concepts can be taught through didactics, practical training remains a challenge.

We present a curriculum tool that was inexpensive and required relatively little preparation to implement. The only materials needed were poster board and paper, with only one facilitator required per group. The number of faculty needed to conduct this exercise was small, especially compared to large-scale simulation drills. Senior residents can also aid in the facilitator role. As we have repeated this exercise multiple times at our institution, we have made small changes to add variations to the residents' experience. These changes were largely in the type of disaster scenario presented. For example, the scenario here involved an explosion, and part of the didactics focused on blast injuries. Another variation of this exercise was conducted in a similar manner. However, the scenario instead involved a building collapse. Concepts related to crush injuries were discussed in the postexercise lecture. This exposed our learners to unique challenges encountered with different types of disasters.

Being that disasters are rare events, this curriculum provided learners with an experience they may not otherwise ever experience. It utilized the theory of andragogy and Kolb's experiential learning as learners were provided a problem and expected to work through the activity with minimal direction from the facilitator.^16–17^ They were then provided the setting to reflect on the experience as a group and identify their errors. To complete the four identified stages of Kolb's experiential learning, learners would need to identify when principles can be learned from this experience, decide if it was meaningful to them, and incorporate it into their existing knowledge. They then needed to apply this new knowledge in a future experience,^17^ which is also why we repeated this exercise multiple times for these learners with a variation on the disaster experience. Many learners may go their entire training or even their entire career without experiencing a real-life disaster. Providing a disaster in the classroom helps to complete this cycle, as well as better prepare residents for independent clinical practice.

One challenge that we faced in reproducing the exercise was preparing a scenario that was appropriate in size for the audience. It was difficult to judge how many mock patients to present to the group as they could be triaged and treated quickly depending on the number and prior experience of participants present. The goal was to make the participants feel overwhelmed by the number of patients and tasks to be completed and feel a limitation in available resources. We recommend erring on the side of having a larger number of mock patients prepared. The facilitator can gauge how quickly the participants are able to triage and treat the patients presented in the scenario and have the ability to increase the number of casualties if needed.

### Limitations

One limitation of this exercise was that the experience of each learner might not be identical, which allowed certain individuals to practice learned skills, while others might miss out. For example, the curriculum began with a lecture introducing topics specific to disaster medicine such as START triage. During the exercise, participants assigned roles and took on different tasks. This may lead to only one or two individuals assigned to triaging patients. While the entire group was exposed to START triage during the introduction lecture, only a select group of learners were practicing this skill. Also, some individuals may not effectively learn through traditional didactic lectures alone. The variation in learner experiences could explain why the medical knowledge scores did not significantly improve from pre- to postsurvey.

The authors' focus for this exercise was centralized more around the process, rather than the medical knowledge gained by individual learners. The ability to assess an individual's disaster readiness was limited to direct observation of participants and subjective evaluation including perceptions and confidence level. A better way to evaluate if this exercise truly prepared an individual could be to follow up with our participants after experiencing a disaster clinically, but this is not feasible as some residents may complete their residency without encountering a disaster experience clinically.

In addition, the structure of EM residency does not allow follow-up of the same learners to be performed easily if this exercise were to be repeated. Residents in EM rotate through different off-service rotations including multiple intensive care units, where EM didactic time is not protected. Additionally, EM residents are permitted to be absent from dedicated didactic time should it interfere with duty hours, such as sessions between night shifts. For these reasons, it would be impossible to ensure that the exact same cohort of residents would experience this exercise at a later date and could participate in a follow-up survey.

Nonetheless, a limited number of responses was a major limitation to our research methods and results. This was also largely due to the small size of our residency program.

### Future Directions

Recognizing the limitation of our small sample size, this exercise and study could be repeated at larger institutions. Our results demonstrated the versatility of this curriculum and potential utility at resource-limited institutions. It can easily be adapted for a variety of departments. The map can be changed to match that of specific departments or other practice milieus. Similarly, the disaster scene map can reflect local areas that may be vulnerable to natural or manmade disasters.

Decay of skills occurs when an individual loses all or part of their ability due to lack of exposure or practice^18^ and is of particular concern for disaster medicine as it is something rarely experienced by an individual. This exercise could be repeated in residency or hospital training on a regular basis in an attempt to address this. While this tool is ideal for curricular development in residency programs, it can also be applied for training all staff in EDs at nonacademic and rural institutions.

This tabletop exercise was adapted for use on a medical mission trip to Mampong, Ghana in October 2019 as part of a 3-day disaster and trauma management course designed for local health care workers. Scenarios were tailored to demonstrate disasters endemic to the area with the appropriate prehospital and hospital capabilities reflected in the exercise. While we did not formally survey the participants on their experience with the tabletop exercise, verbally they gave positive feedback and enjoyed the hands on, practical aspect of the experience. Having both physicians and nurses in teams also allowed for a more realistic and collaborative approach to the scenarios. The materials used, which consisted largely of poster board and colored index cards, were prepared prior to departure to Ghana, and transported easily in the luggage of volunteer staff. Due to the modest investment and flexible scope, participants were optimistic that they could continue conducting similar tabletop exercises within their own institutions. Overall, the tabletop exercise was easily translated and our experience in Ghana demonstrated its adaptability for health care professionals in different settings with variable resources.

### Conclusion

Strong responses from the residents in improving their confidence and ability to encounter similar disaster scenarios in the future support its use for implementation into residency curricula. This disaster preparedness training has the potential to be expanded outside of residency teaching for collaborate staff training in EDs, as it was used in Ghana. This exercise is low cost, easy to reproduce, and can be adapted for many types of disasters that could threaten a local community.

## Appendices

Exercise Lecture.pptxDisaster Scene Packet.docxHospital Scene Packet.docxPre-Exercise Survey.docxPostexercise Survey.docx
All appendices are peer reviewed as integral parts of the Original Publication.
