# Supplementary material for: Disaster Preparedness Training for Emergency Medicine Residents Using a Tabletop Exercise
Source: MedEdPORTAL. 2021 Mar 12;17:11119. doi: 10.15766/mep_2374-8265.11119 (PMC7970644; doi:10.15766/mep_2374-8265.11119)
Supplement: Supplementary file 1 — Exercise Lecture.pptxDisaster Scene Packet.docxHospital Scene Packet.docxPre-Exercise Survey.docxPostexercise Survey.docx [file mep_2374-8265.11119-s001.zip › A. Exercise Lecture.pptx]

## Slide 1
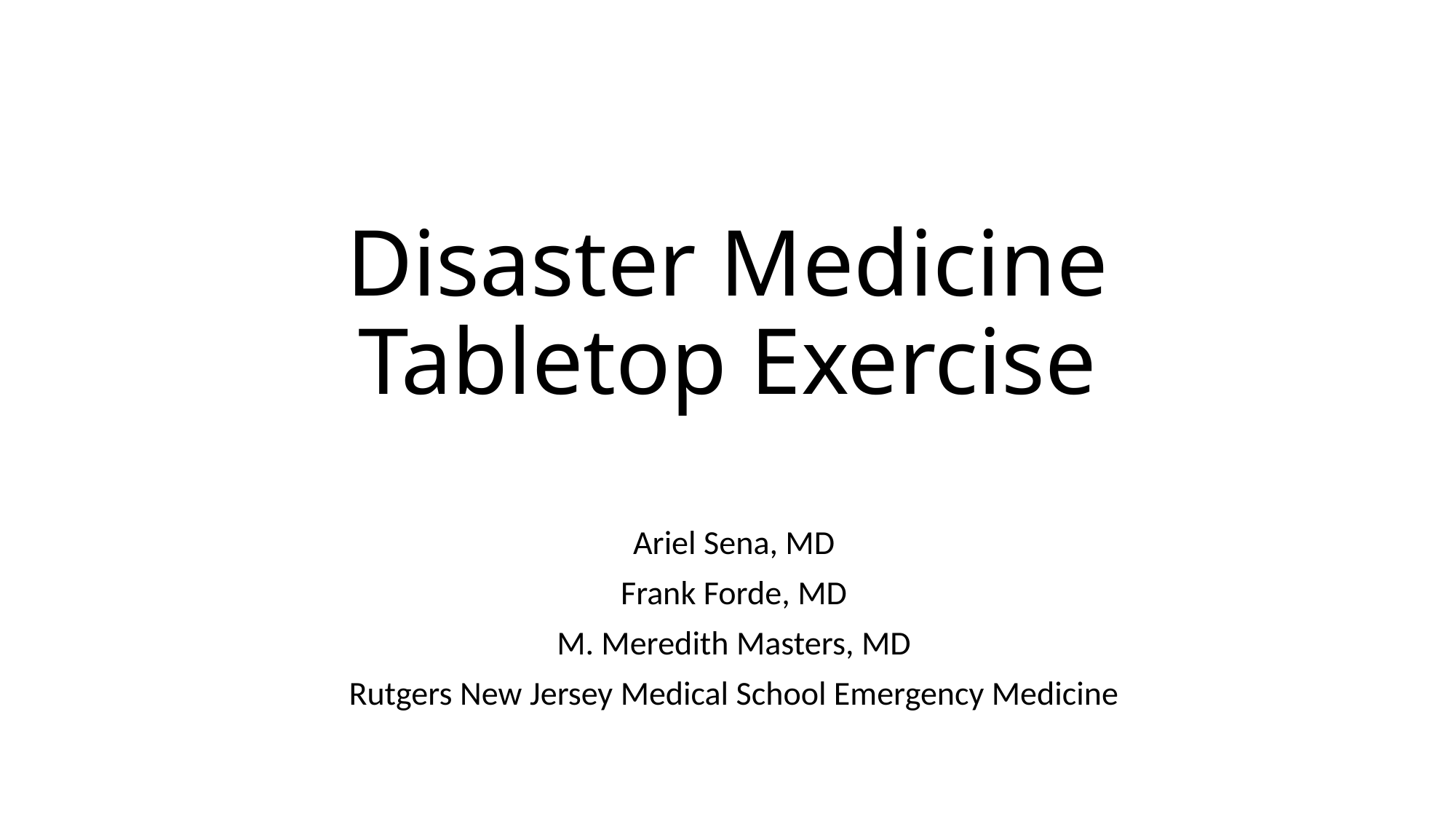

# Disaster Medicine Tabletop Exercise
Ariel Sena, MD
Frank Forde, MD
M. Meredith Masters, MD
Rutgers New Jersey Medical School Emergency Medicine

## Slide 2
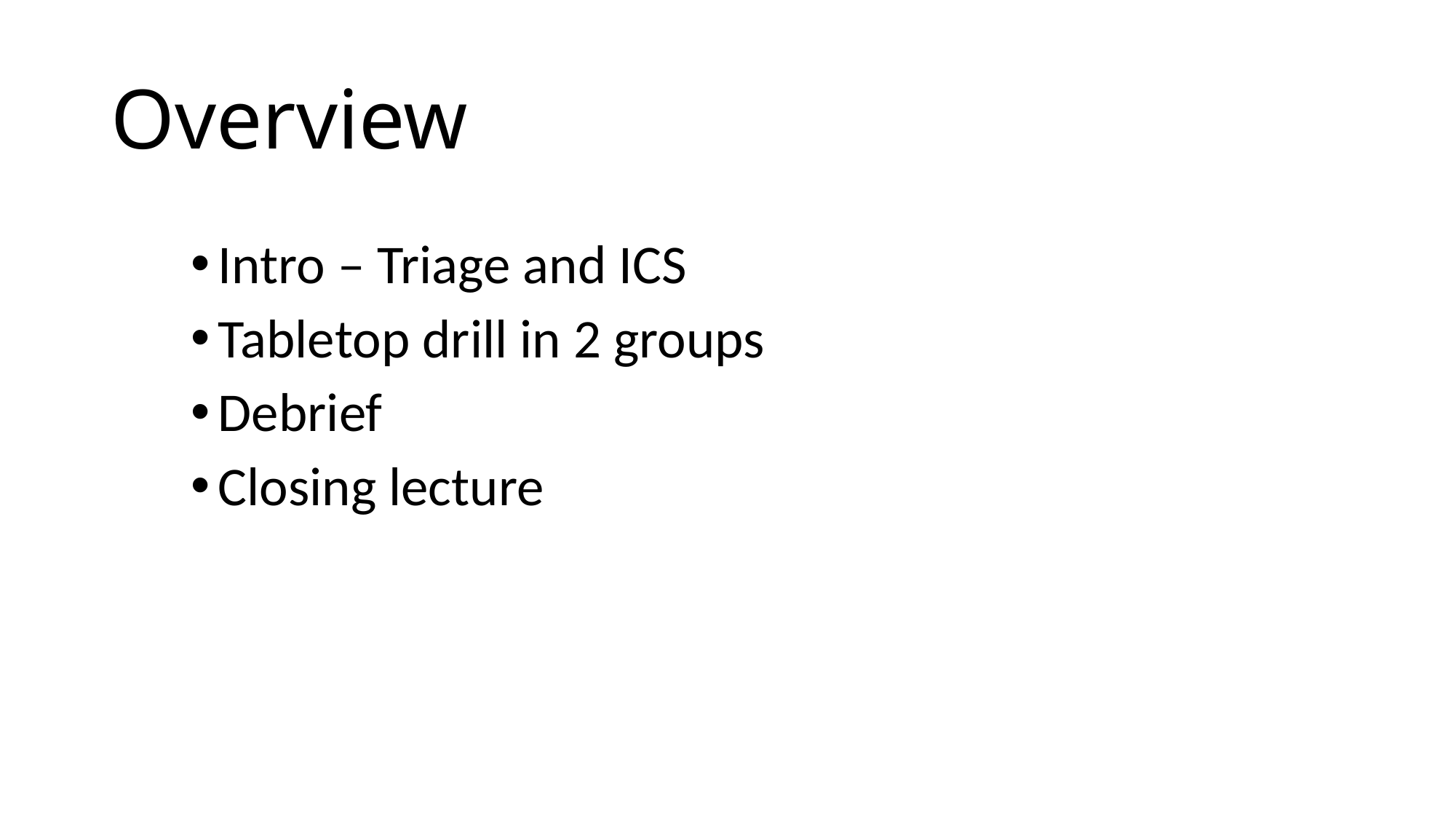

# Overview
Intro – Triage and ICS
Tabletop drill in 2 groups
Debrief
Closing lecture

## Slide 3
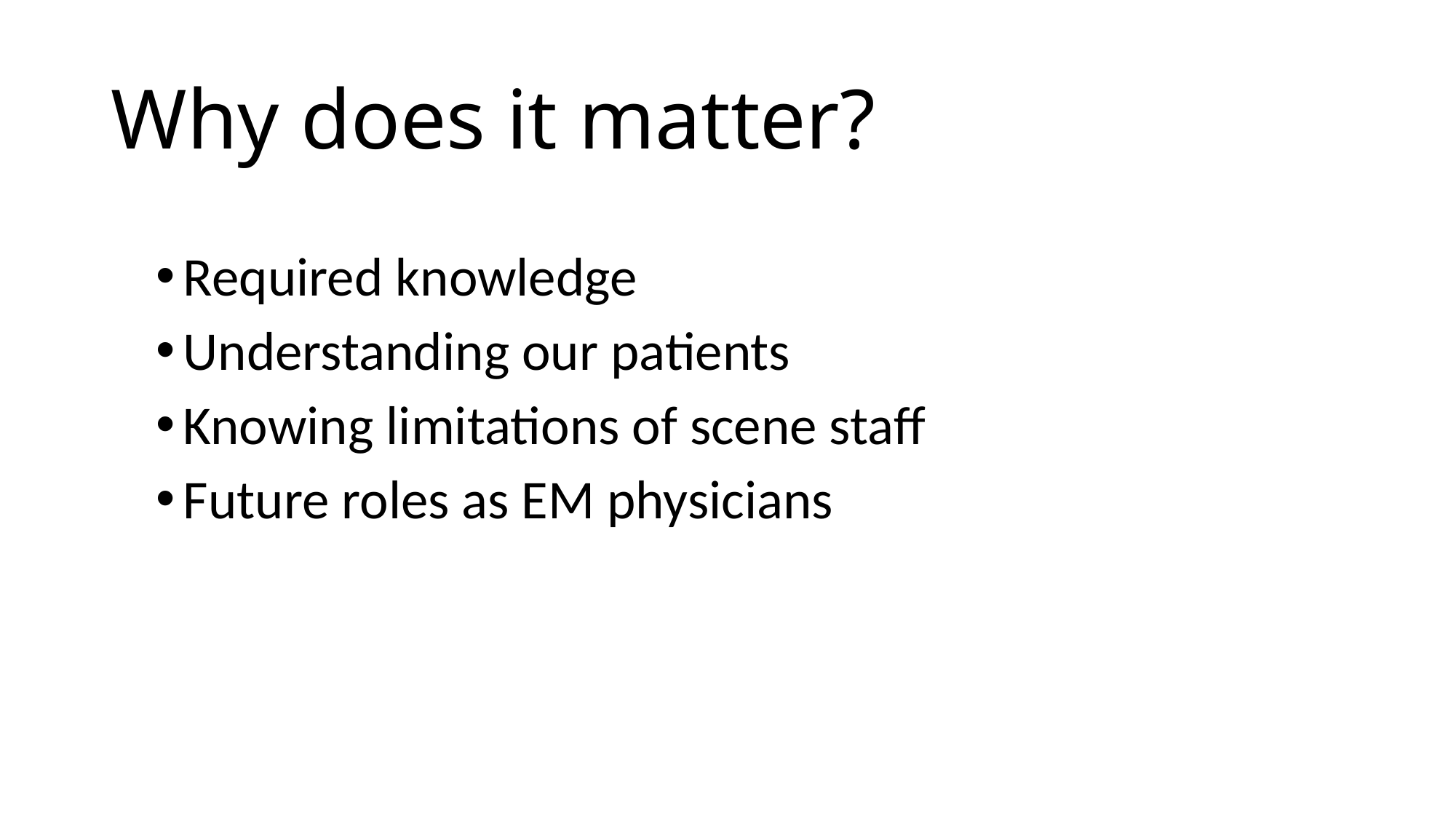

# Why does it matter?
Required knowledge
Understanding our patients
Knowing limitations of scene staff
Future roles as EM physicians

## Slide 4
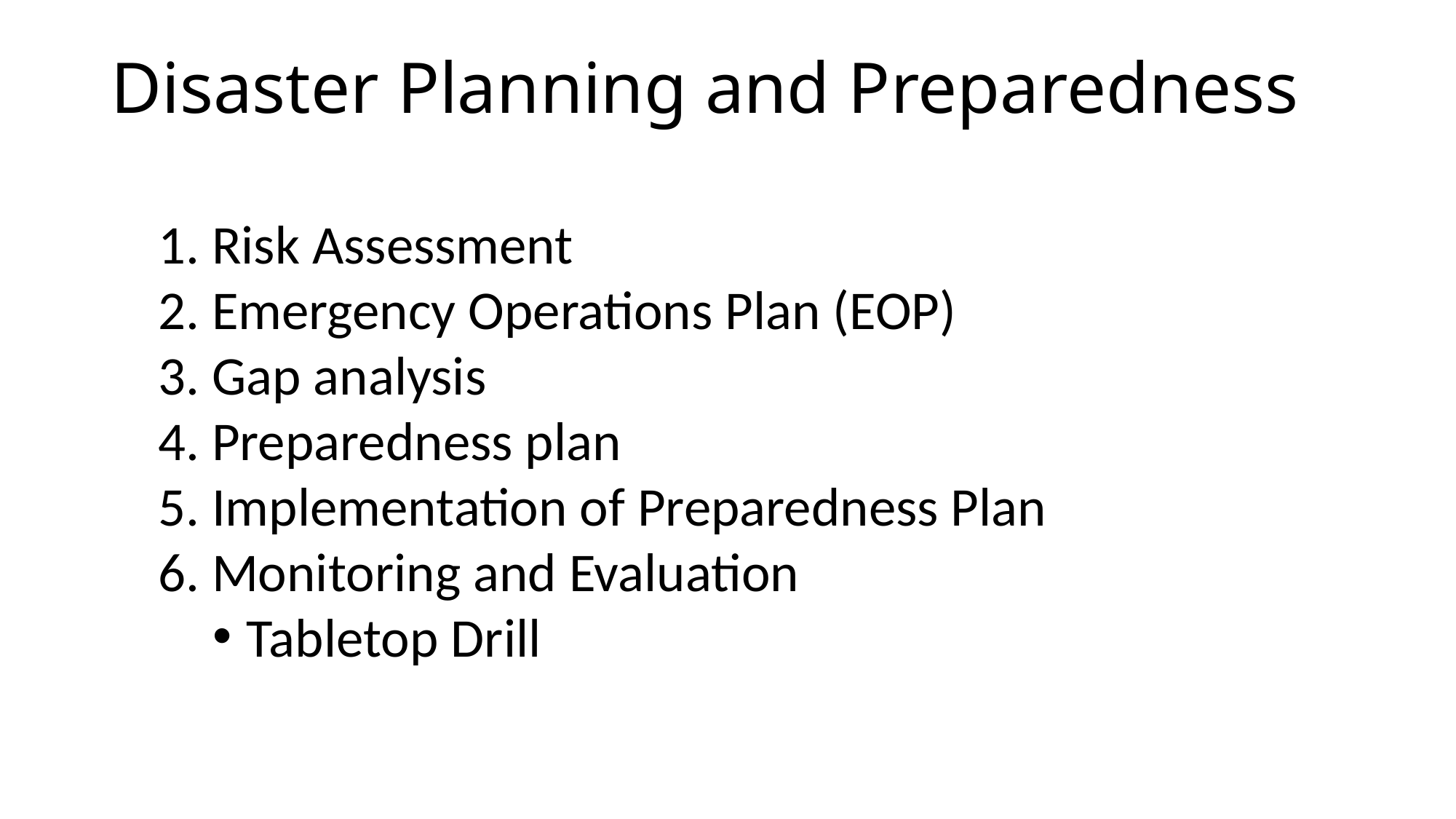

# Disaster Planning and Preparedness
 Risk Assessment
 Emergency Operations Plan (EOP)
 Gap analysis
 Preparedness plan
 Implementation of Preparedness Plan
 Monitoring and Evaluation
Tabletop Drill

## Slide 5
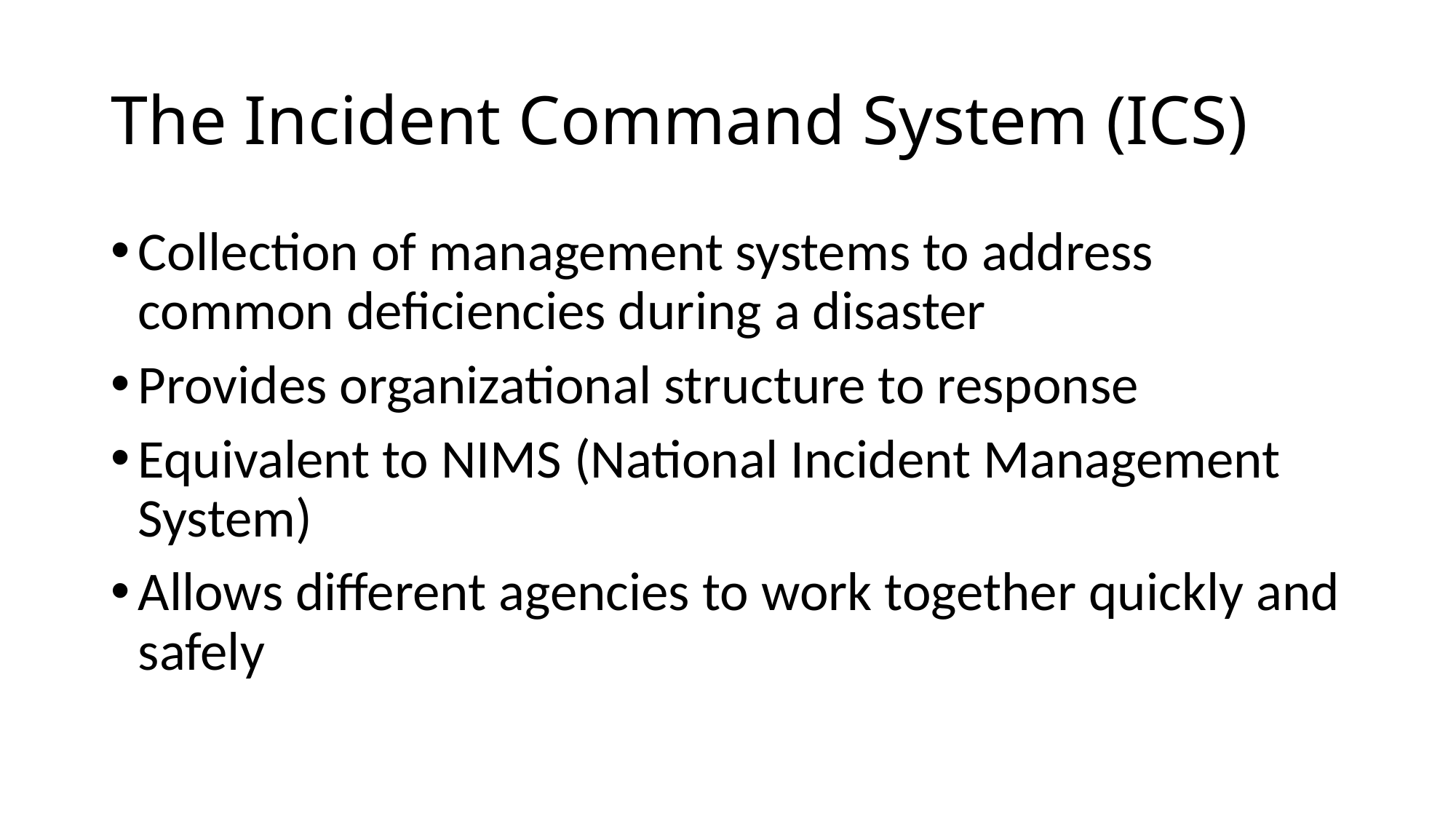

# The Incident Command System (ICS)
Collection of management systems to address common deficiencies during a disaster
Provides organizational structure to response
Equivalent to NIMS (National Incident Management System)
Allows different agencies to work together quickly and safely

## Slide 6
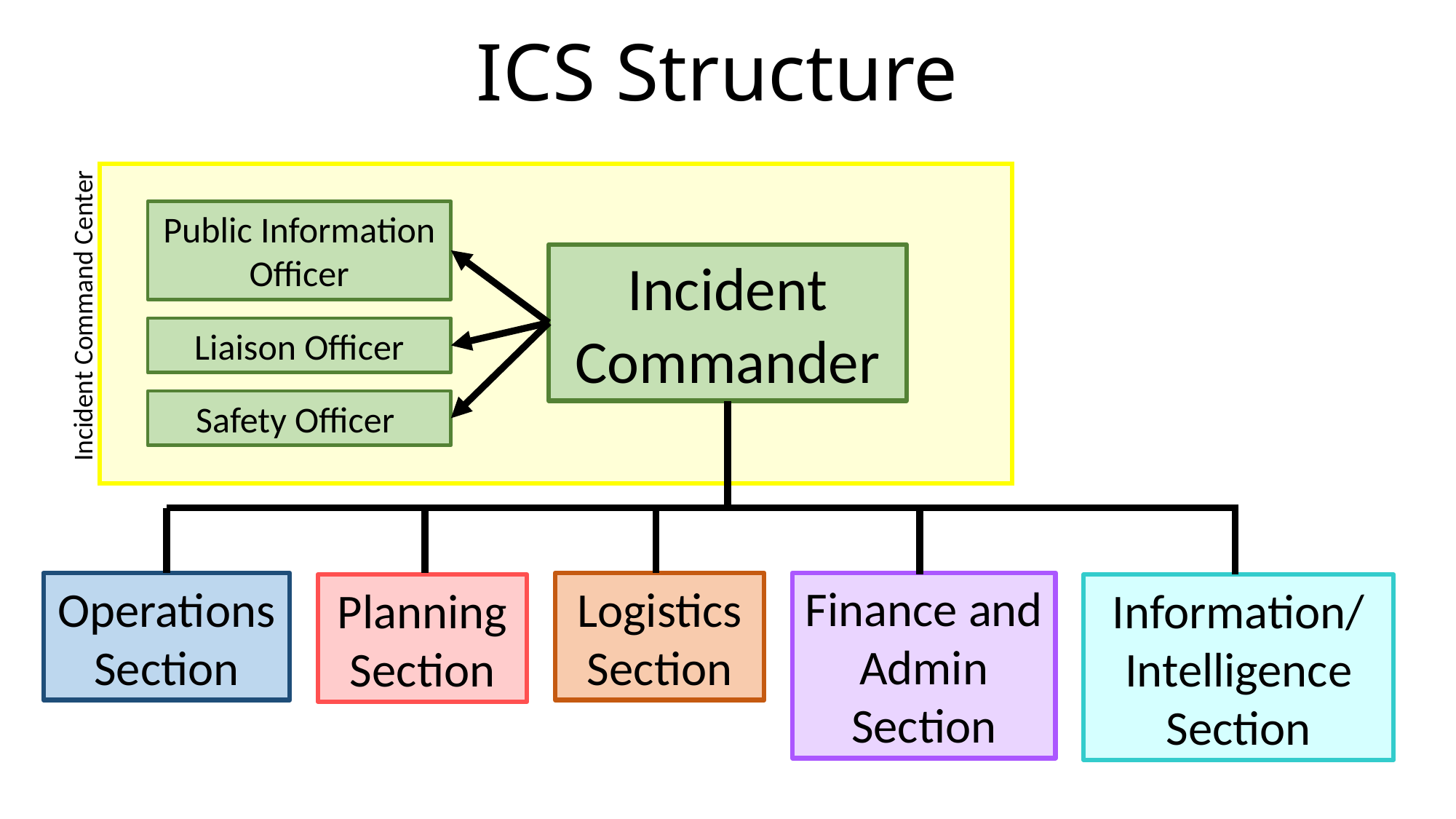

# ICS Structure
Public Information Officer
Incident Commander
Incident Command Center
Liaison Officer
Safety Officer
Finance and Admin Section
Operations Section
Logistics Section
Planning Section
Information/ Intelligence Section

## Slide 7
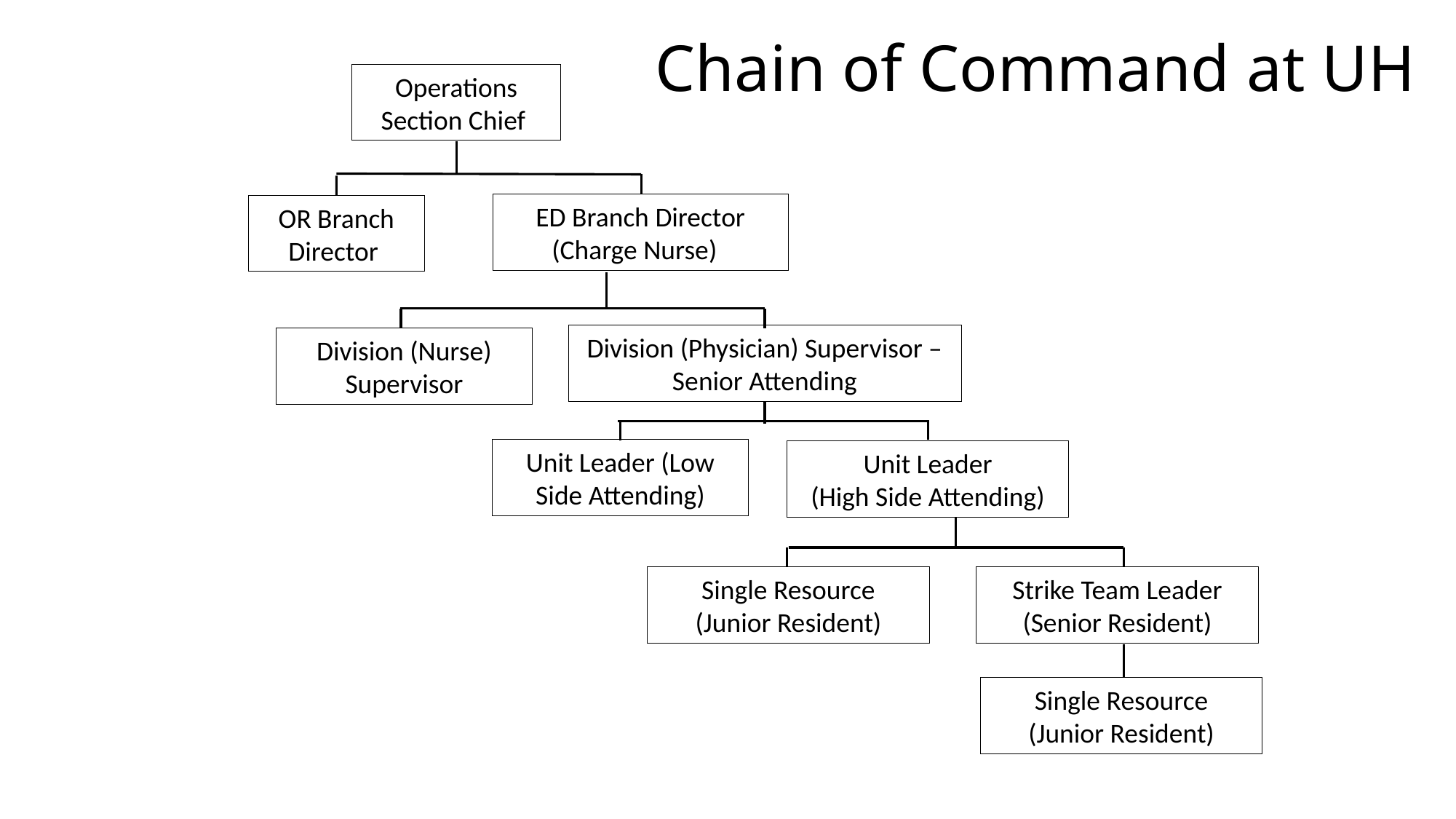

# Chain of Command at UH
Operations Section Chief
ED Branch Director (Charge Nurse)
OR Branch Director
Division (Physician) Supervisor – Senior Attending
Division (Nurse) Supervisor
Unit Leader (Low Side Attending)
Unit Leader
(High Side Attending)
Strike Team Leader
(Senior Resident)
Single Resource
(Junior Resident)
Single Resource
(Junior Resident)

## Slide 8
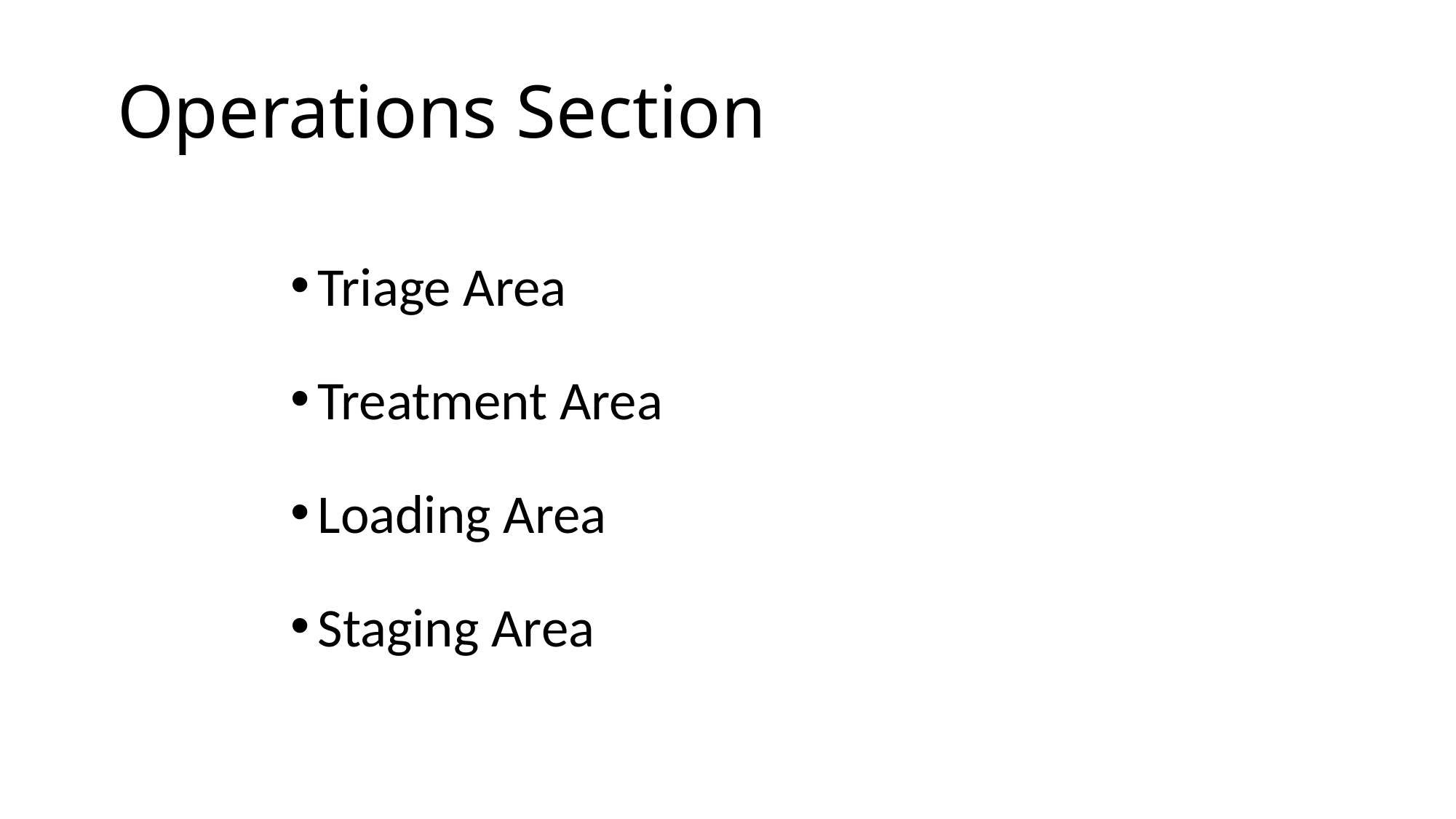

# Operations Section
Triage Area
Treatment Area
Loading Area
Staging Area

## Slide 9
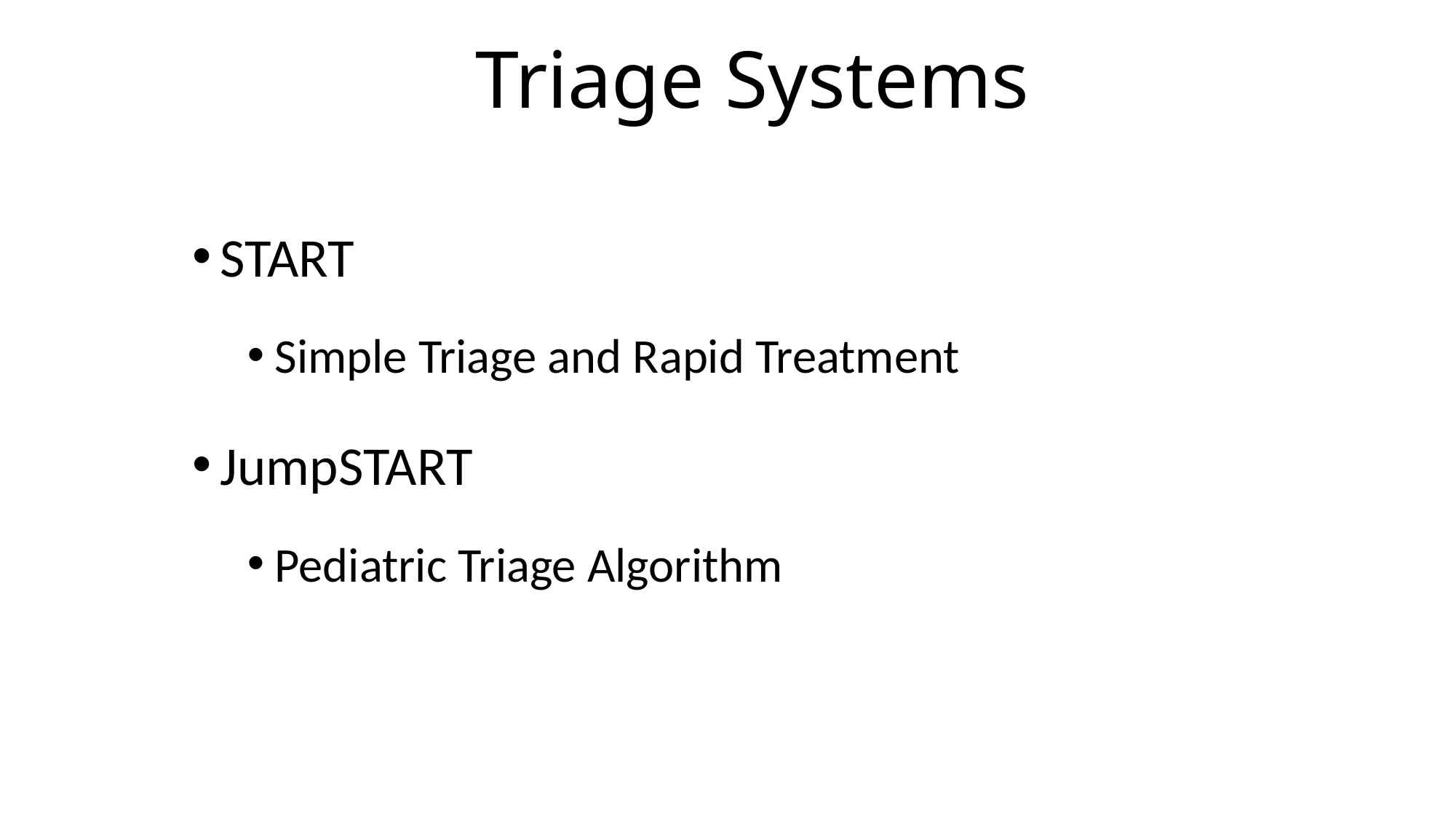

# Triage Systems
START
Simple Triage and Rapid Treatment
JumpSTART
Pediatric Triage Algorithm

## Slide 10
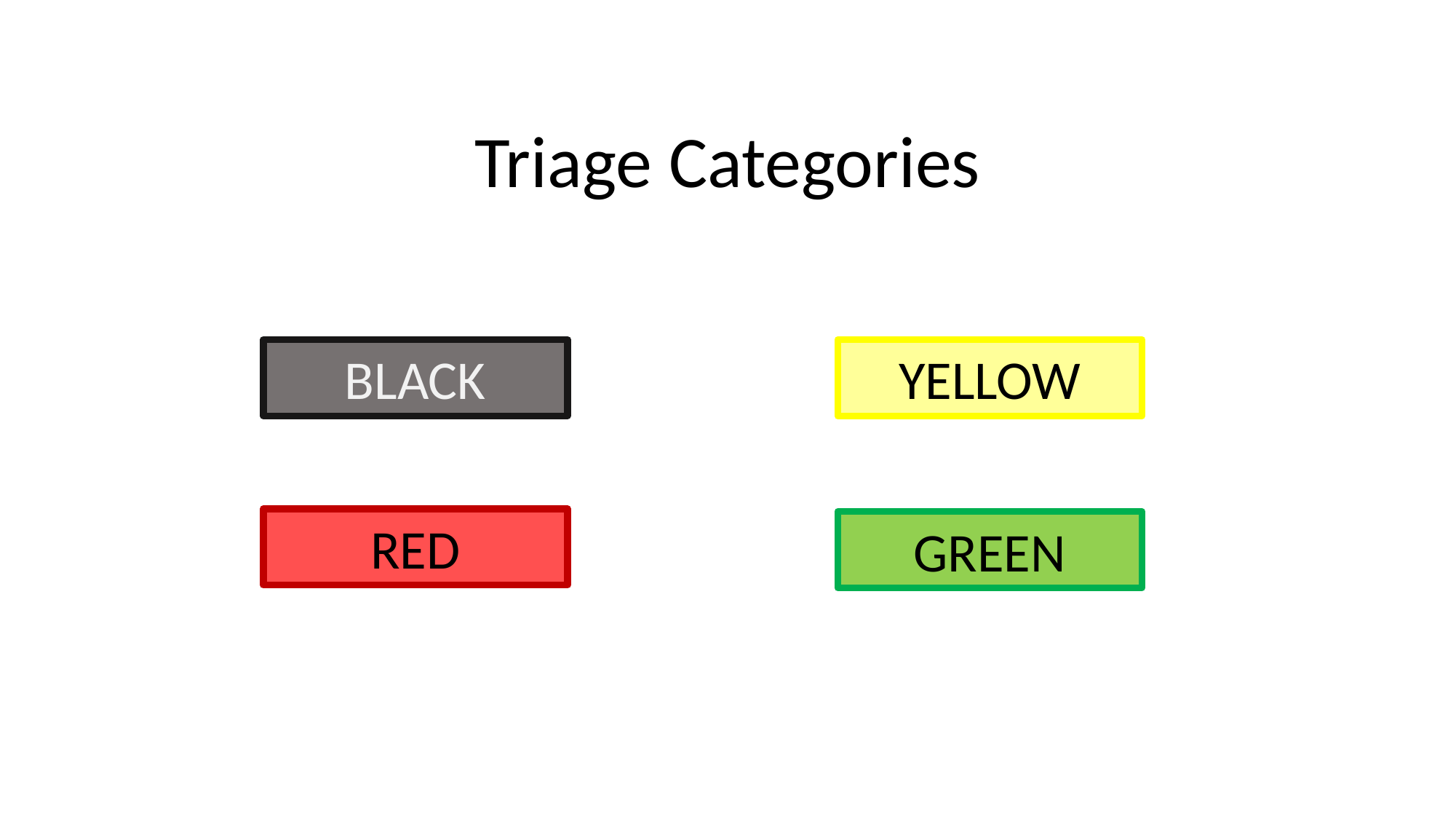

Triage Categories
BLACK
YELLOW
RED
GREEN

## Slide 11
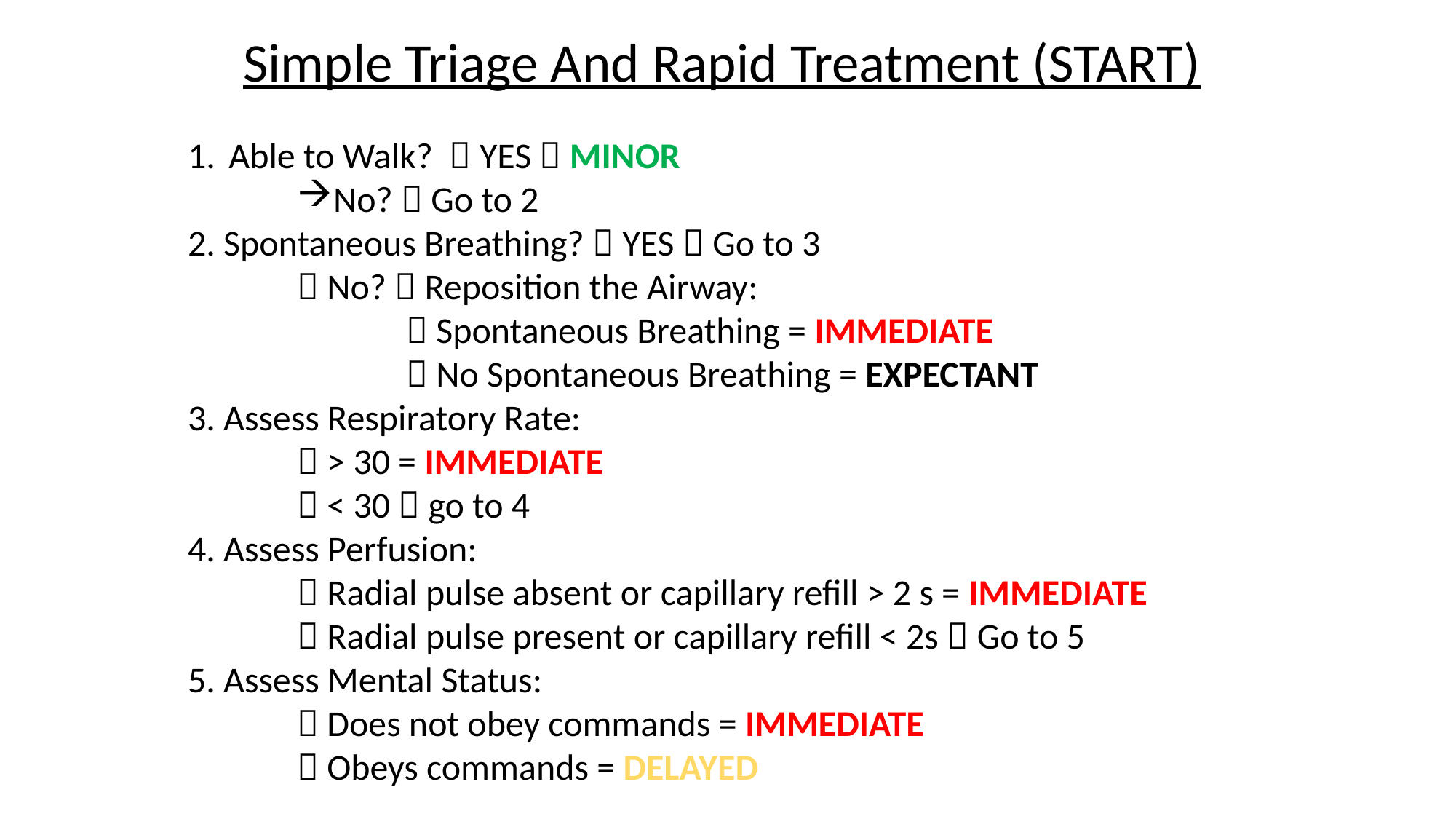

Simple Triage And Rapid Treatment (START)
Able to Walk?  YES  MINOR
No?  Go to 2
2. Spontaneous Breathing?  YES  Go to 3
	 No?  Reposition the Airway:
		 Spontaneous Breathing = IMMEDIATE
		 No Spontaneous Breathing = EXPECTANT
3. Assess Respiratory Rate:
	 > 30 = IMMEDIATE
	 < 30  go to 4
4. Assess Perfusion:
	 Radial pulse absent or capillary refill > 2 s = IMMEDIATE
	 Radial pulse present or capillary refill < 2s  Go to 5
5. Assess Mental Status:
	 Does not obey commands = IMMEDIATE
	 Obeys commands = DELAYED

## Slide 12
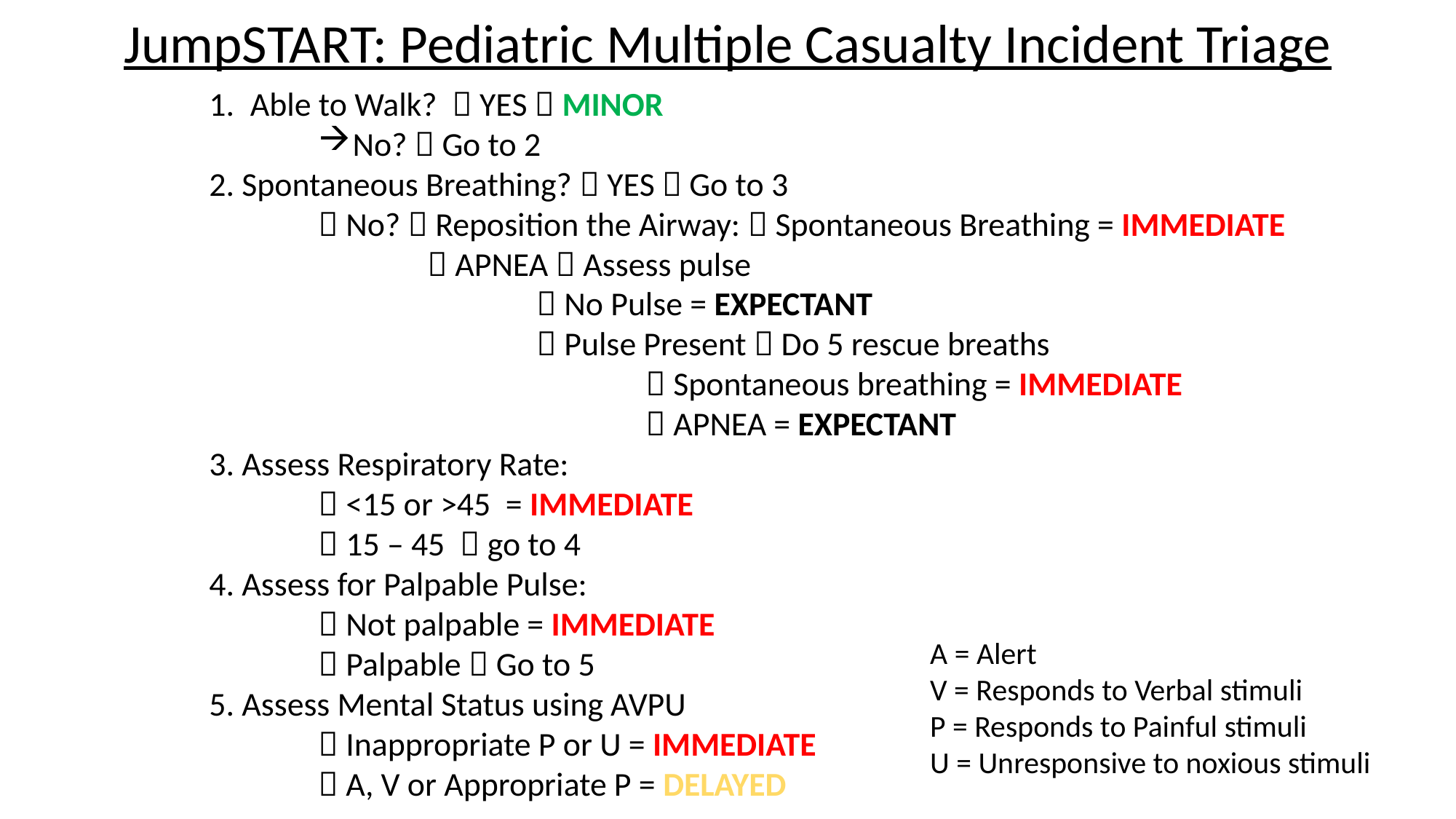

JumpSTART: Pediatric Multiple Casualty Incident Triage
Able to Walk?  YES  MINOR
No?  Go to 2
2. Spontaneous Breathing?  YES  Go to 3
	 No?  Reposition the Airway:  Spontaneous Breathing = IMMEDIATE
		 APNEA  Assess pulse
			 No Pulse = EXPECTANT
			 Pulse Present  Do 5 rescue breaths
				 Spontaneous breathing = IMMEDIATE
				 APNEA = EXPECTANT
3. Assess Respiratory Rate:
	 <15 or >45 = IMMEDIATE
	 15 – 45  go to 4
4. Assess for Palpable Pulse:
	 Not palpable = IMMEDIATE
	 Palpable  Go to 5
5. Assess Mental Status using AVPU
	 Inappropriate P or U = IMMEDIATE
	 A, V or Appropriate P = DELAYED
A = Alert
V = Responds to Verbal stimuli
P = Responds to Painful stimuli
U = Unresponsive to noxious stimuli

## Slide 13
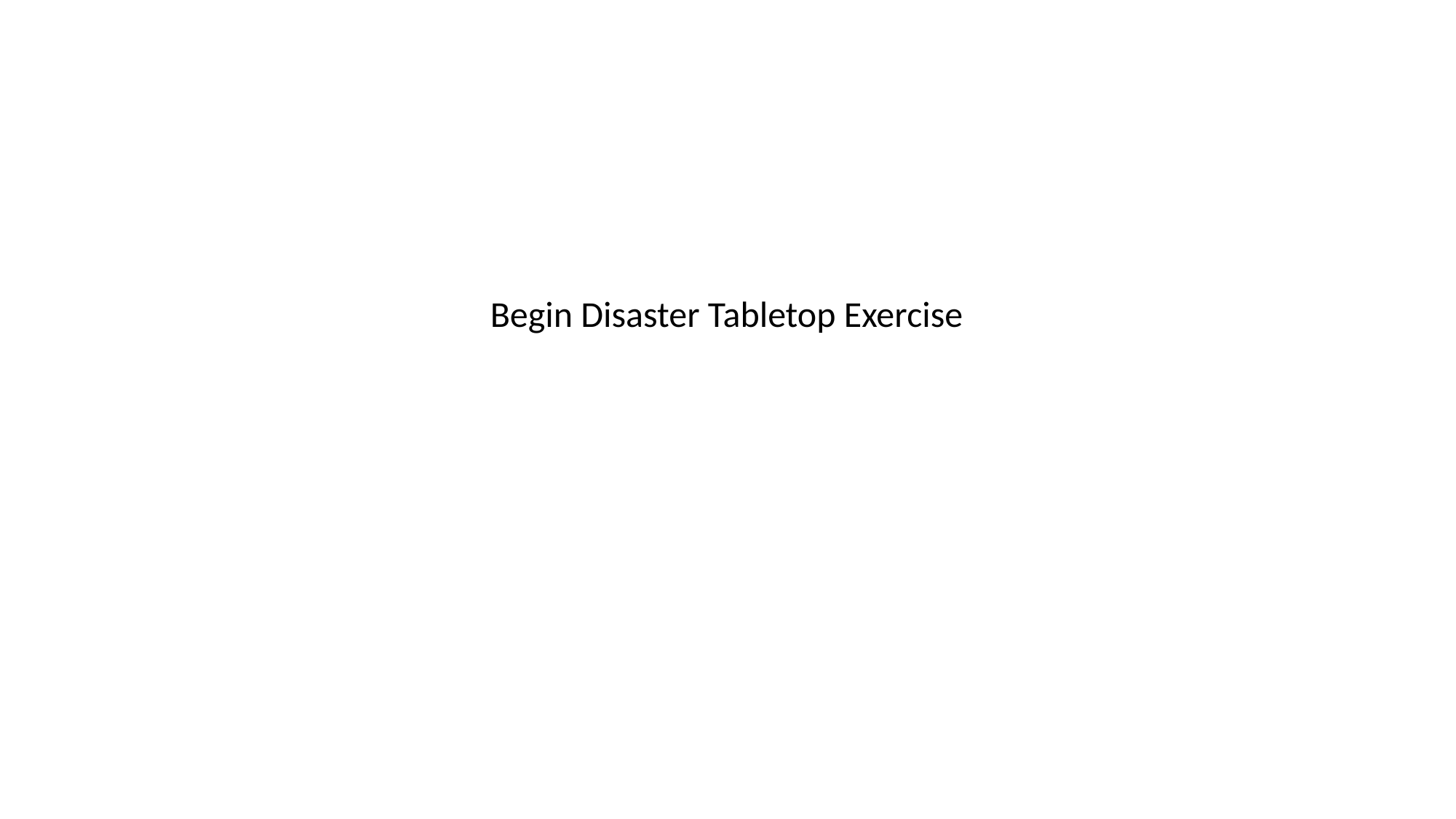

Begin Disaster Tabletop Exercise

## Slide 14
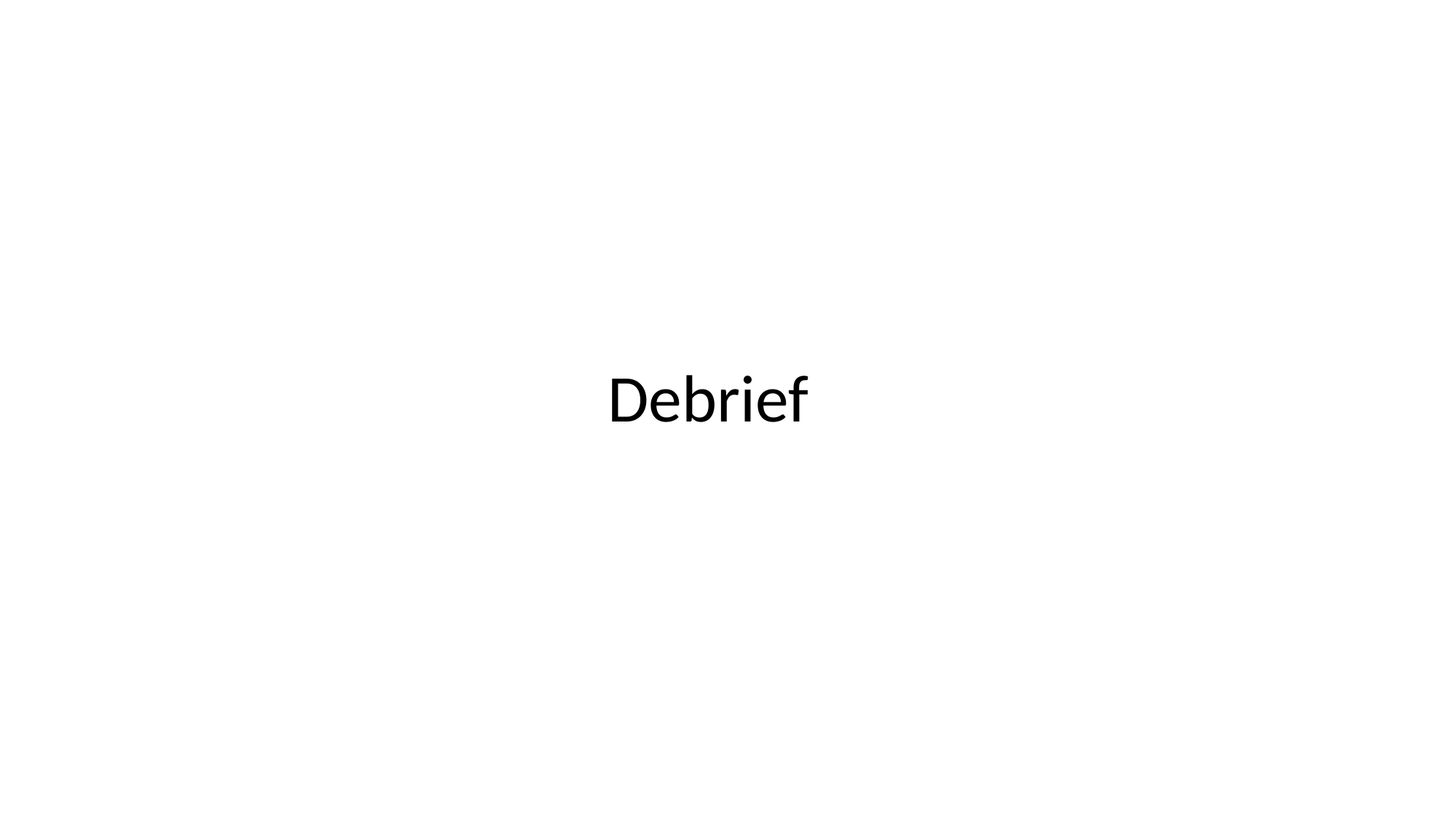

Debrief

## Slide 15
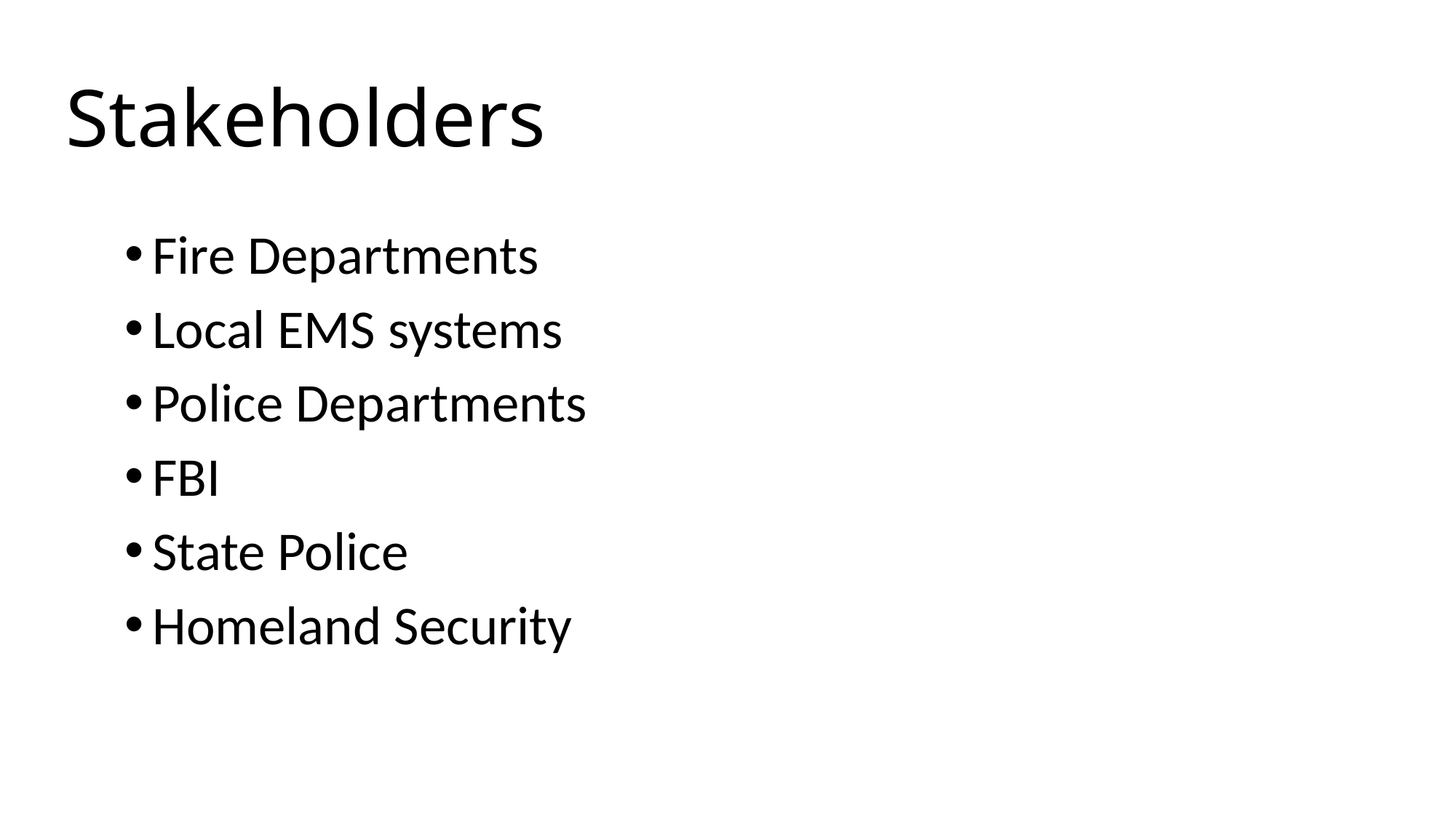

# Stakeholders
Fire Departments
Local EMS systems
Police Departments
FBI
State Police
Homeland Security

## Slide 16
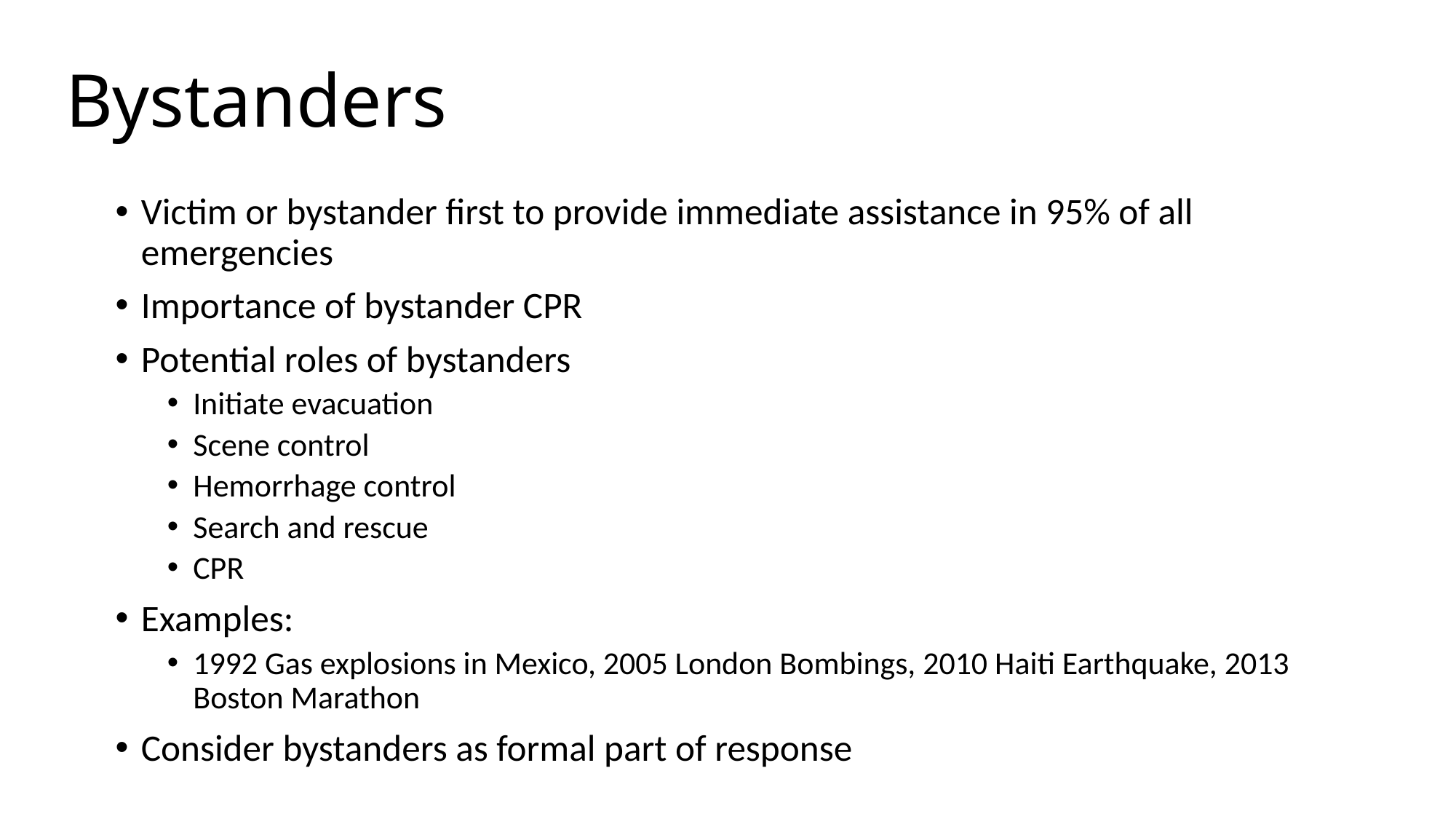

# Bystanders
Victim or bystander first to provide immediate assistance in 95% of all emergencies
Importance of bystander CPR
Potential roles of bystanders
Initiate evacuation
Scene control
Hemorrhage control
Search and rescue
CPR
Examples:
1992 Gas explosions in Mexico, 2005 London Bombings, 2010 Haiti Earthquake, 2013 Boston Marathon
Consider bystanders as formal part of response

## Slide 17
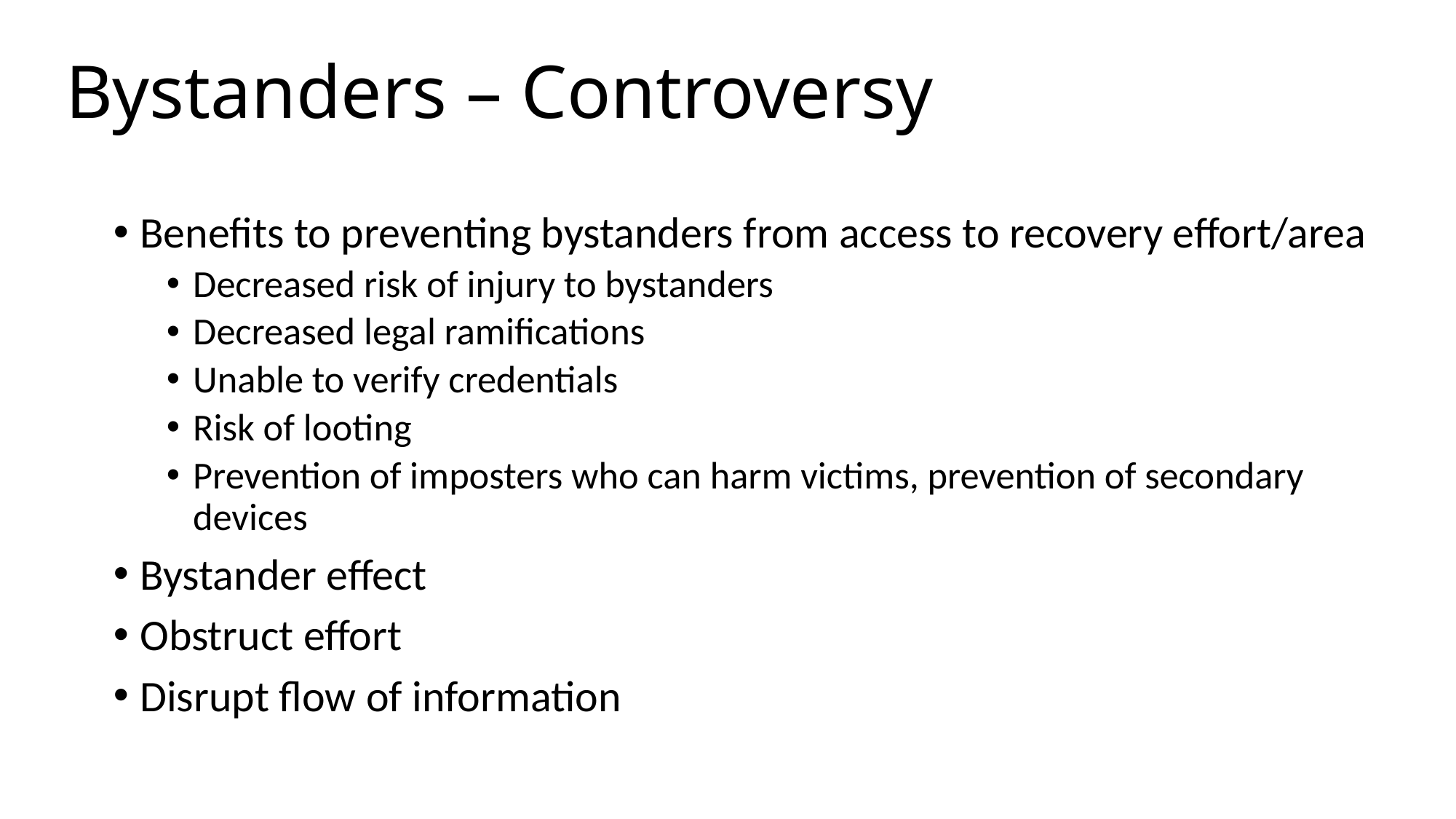

# Bystanders – Controversy
Benefits to preventing bystanders from access to recovery effort/area
Decreased risk of injury to bystanders
Decreased legal ramifications
Unable to verify credentials
Risk of looting
Prevention of imposters who can harm victims, prevention of secondary devices
Bystander effect
Obstruct effort
Disrupt flow of information

## Slide 18
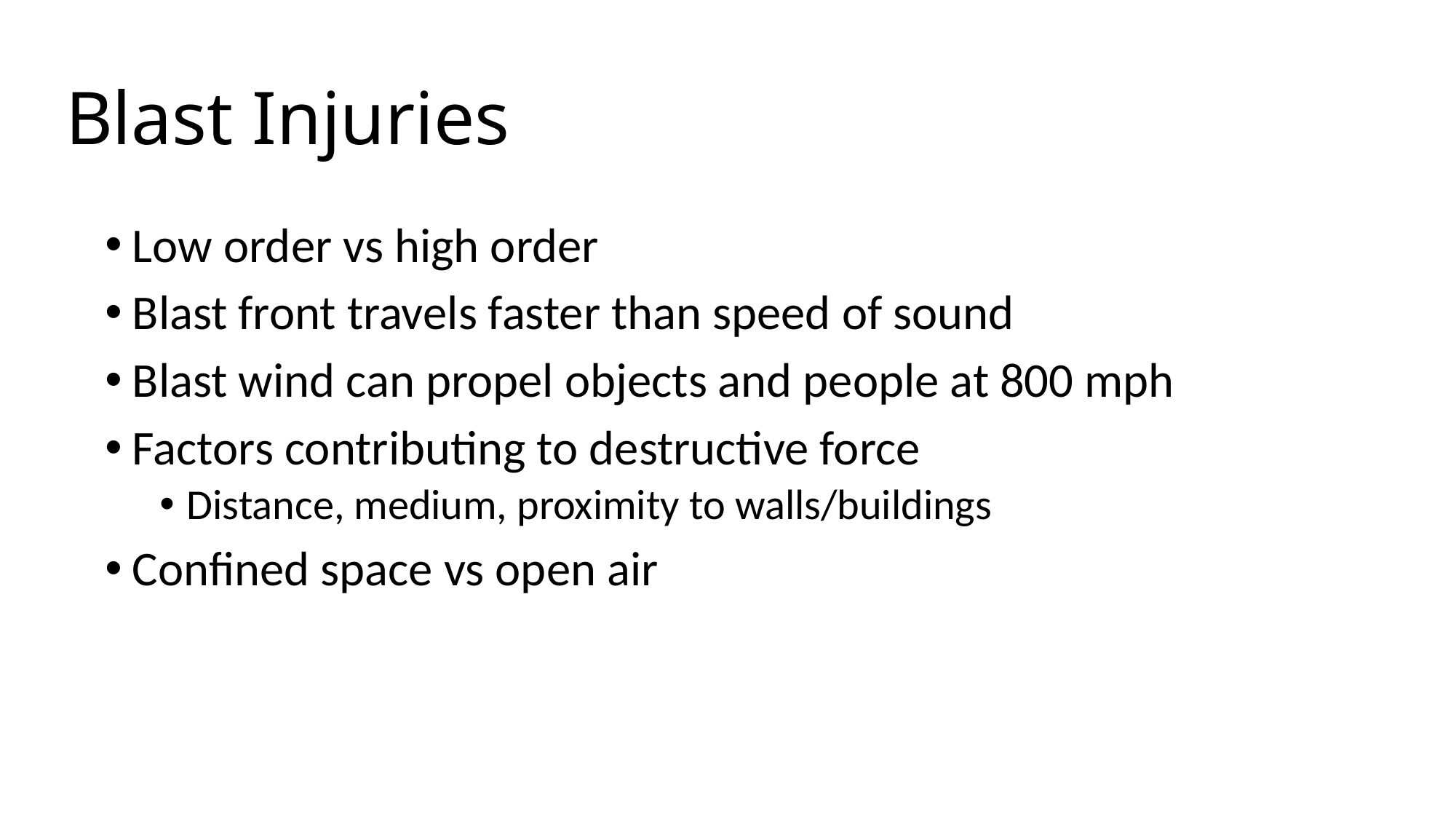

# Blast Injuries
Low order vs high order
Blast front travels faster than speed of sound
Blast wind can propel objects and people at 800 mph
Factors contributing to destructive force
Distance, medium, proximity to walls/buildings
Confined space vs open air

## Slide 19
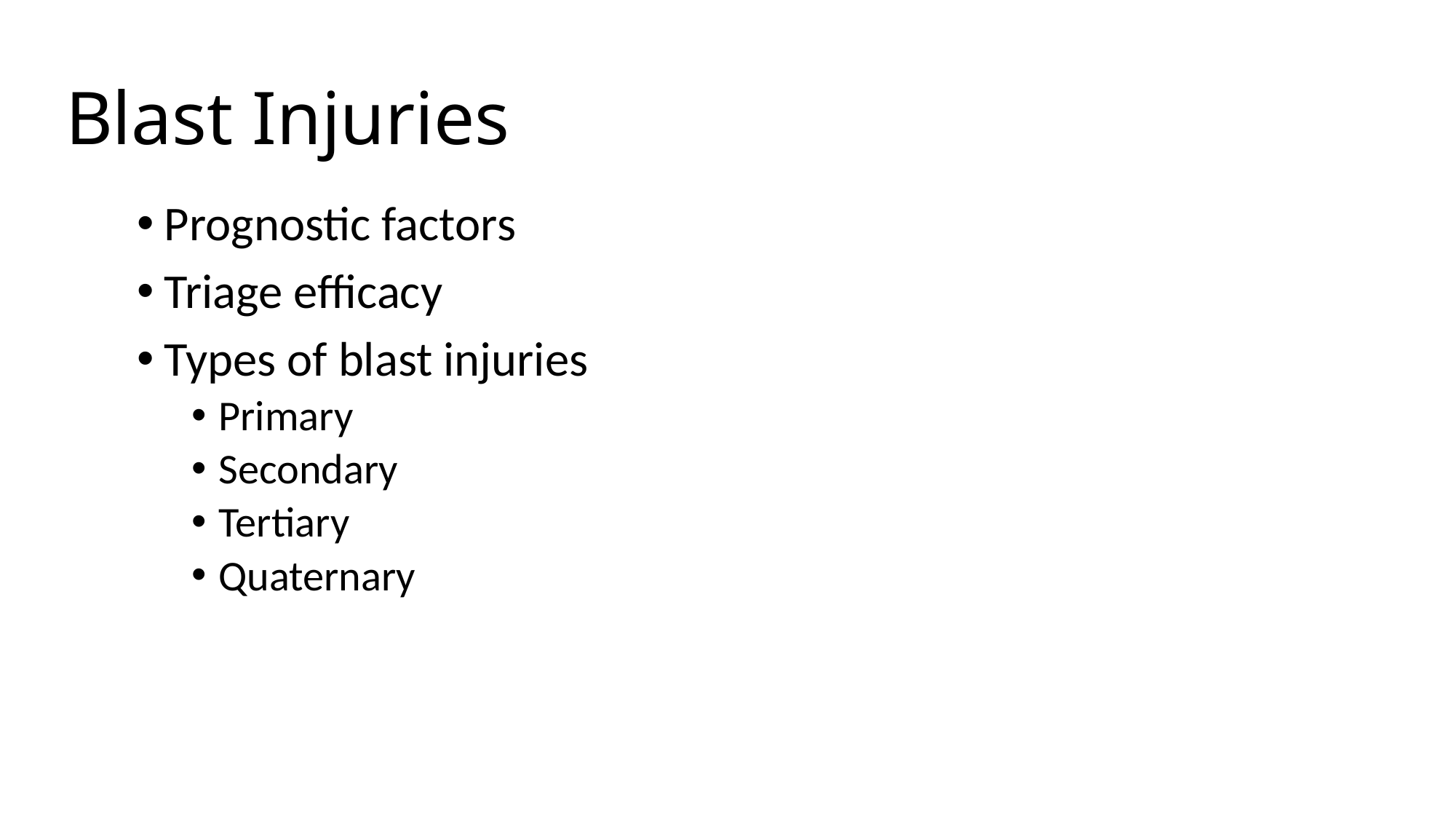

# Blast Injuries
Prognostic factors
Triage efficacy
Types of blast injuries
Primary
Secondary
Tertiary
Quaternary

## Slide 20
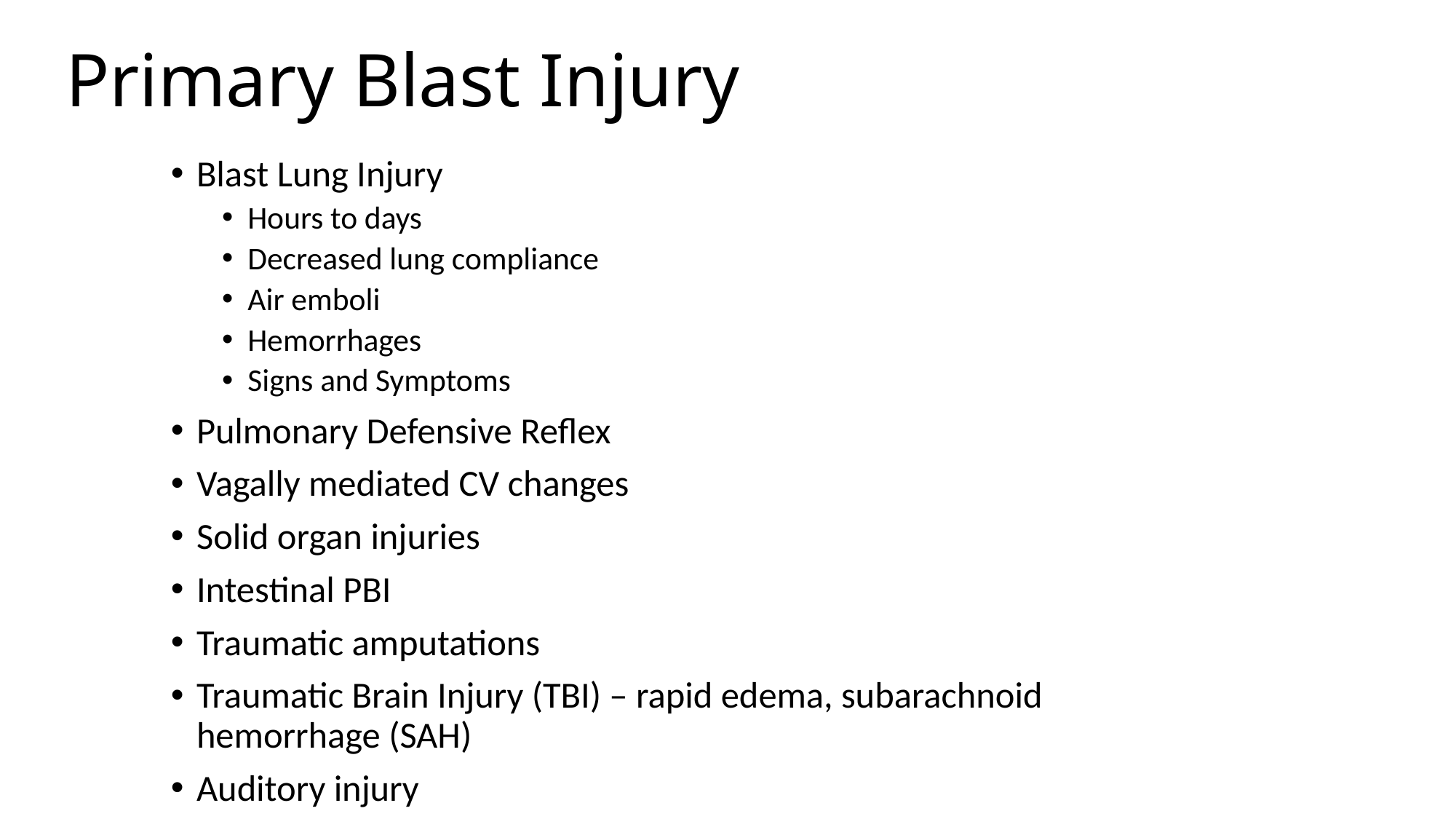

# Primary Blast Injury
Blast Lung Injury
Hours to days
Decreased lung compliance
Air emboli
Hemorrhages
Signs and Symptoms
Pulmonary Defensive Reflex
Vagally mediated CV changes
Solid organ injuries
Intestinal PBI
Traumatic amputations
Traumatic Brain Injury (TBI) – rapid edema, subarachnoid hemorrhage (SAH)
Auditory injury

## Slide 21
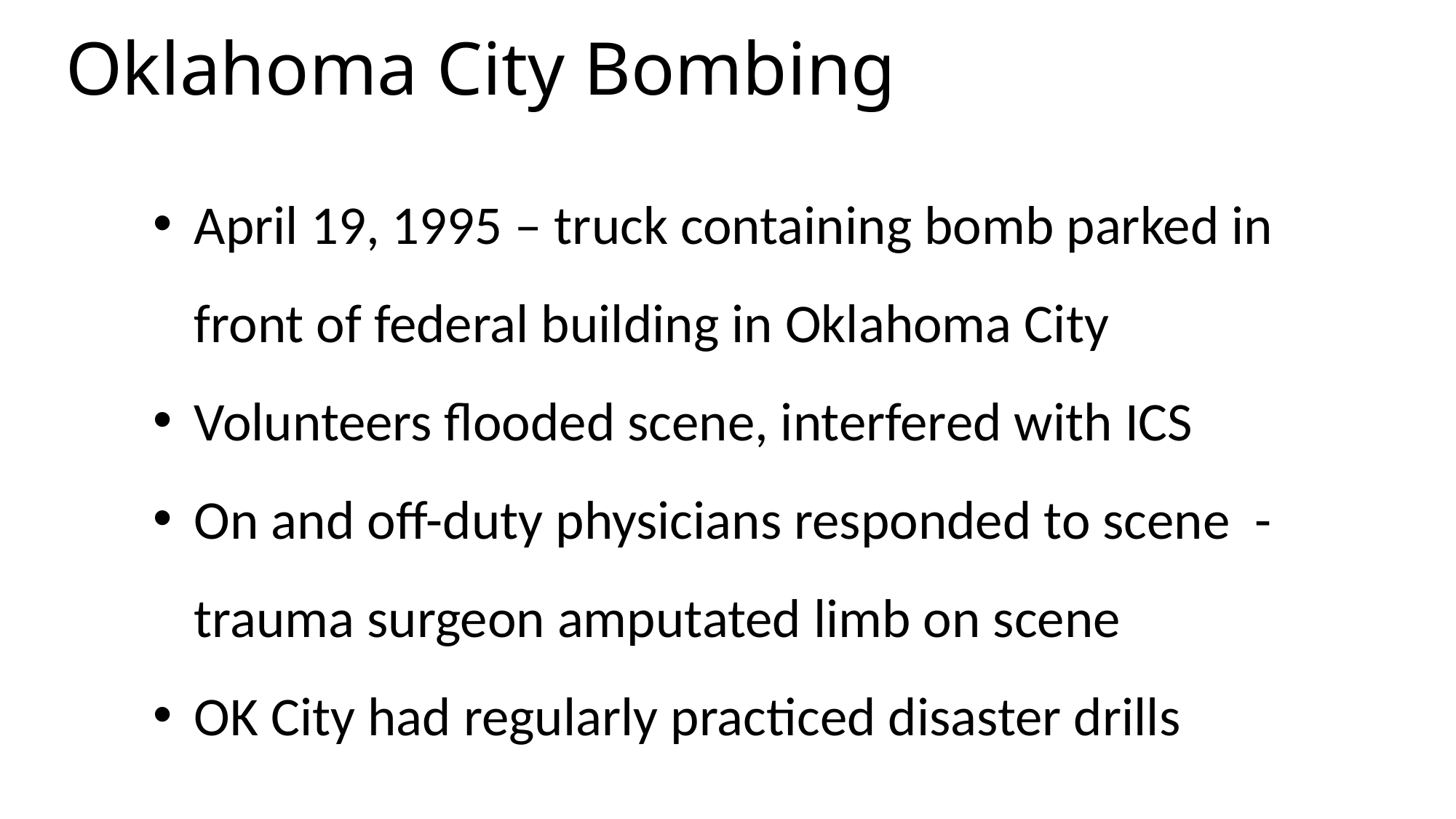

# Oklahoma City Bombing
April 19, 1995 – truck containing bomb parked in front of federal building in Oklahoma City
Volunteers flooded scene, interfered with ICS
On and off-duty physicians responded to scene - trauma surgeon amputated limb on scene
OK City had regularly practiced disaster drills

## Slide 22
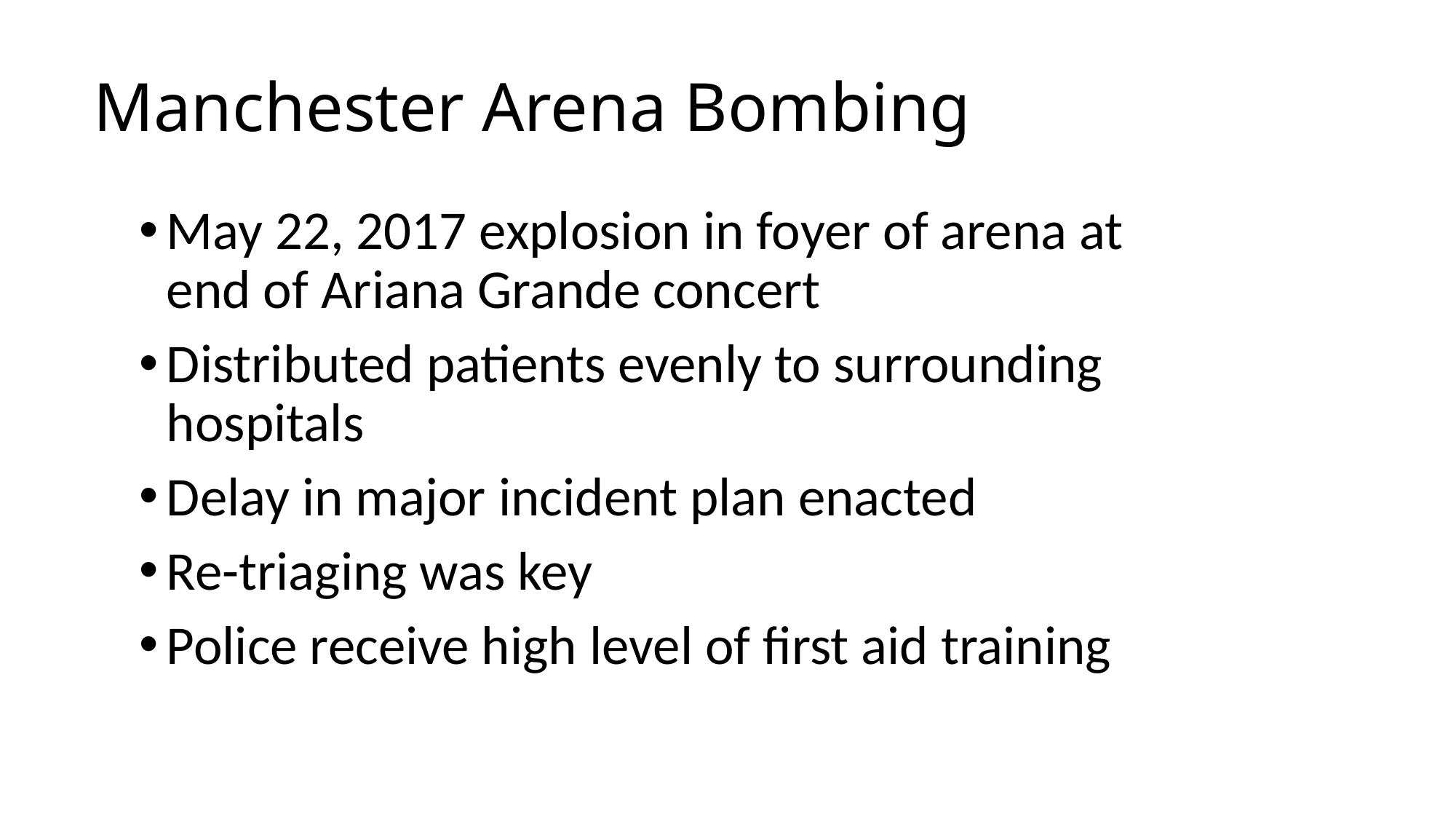

# Manchester Arena Bombing
May 22, 2017 explosion in foyer of arena at end of Ariana Grande concert
Distributed patients evenly to surrounding hospitals
Delay in major incident plan enacted
Re-triaging was key
Police receive high level of first aid training

## Slide 23
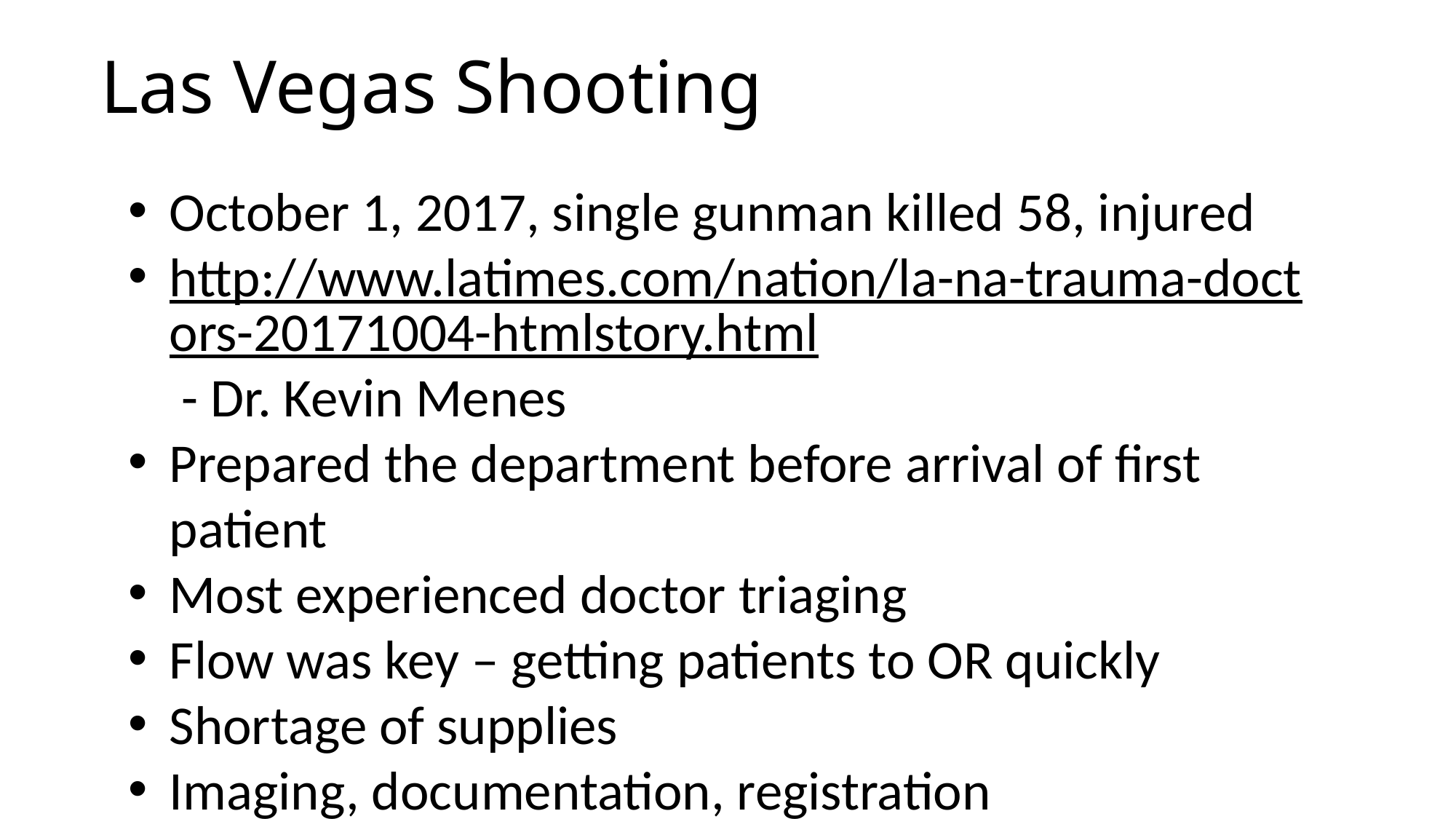

# Las Vegas Shooting
October 1, 2017, single gunman killed 58, injured
http://www.latimes.com/nation/la-na-trauma-doctors-20171004-htmlstory.html - Dr. Kevin Menes
Prepared the department before arrival of first patient
Most experienced doctor triaging
Flow was key – getting patients to OR quickly
Shortage of supplies
Imaging, documentation, registration

## Slide 24
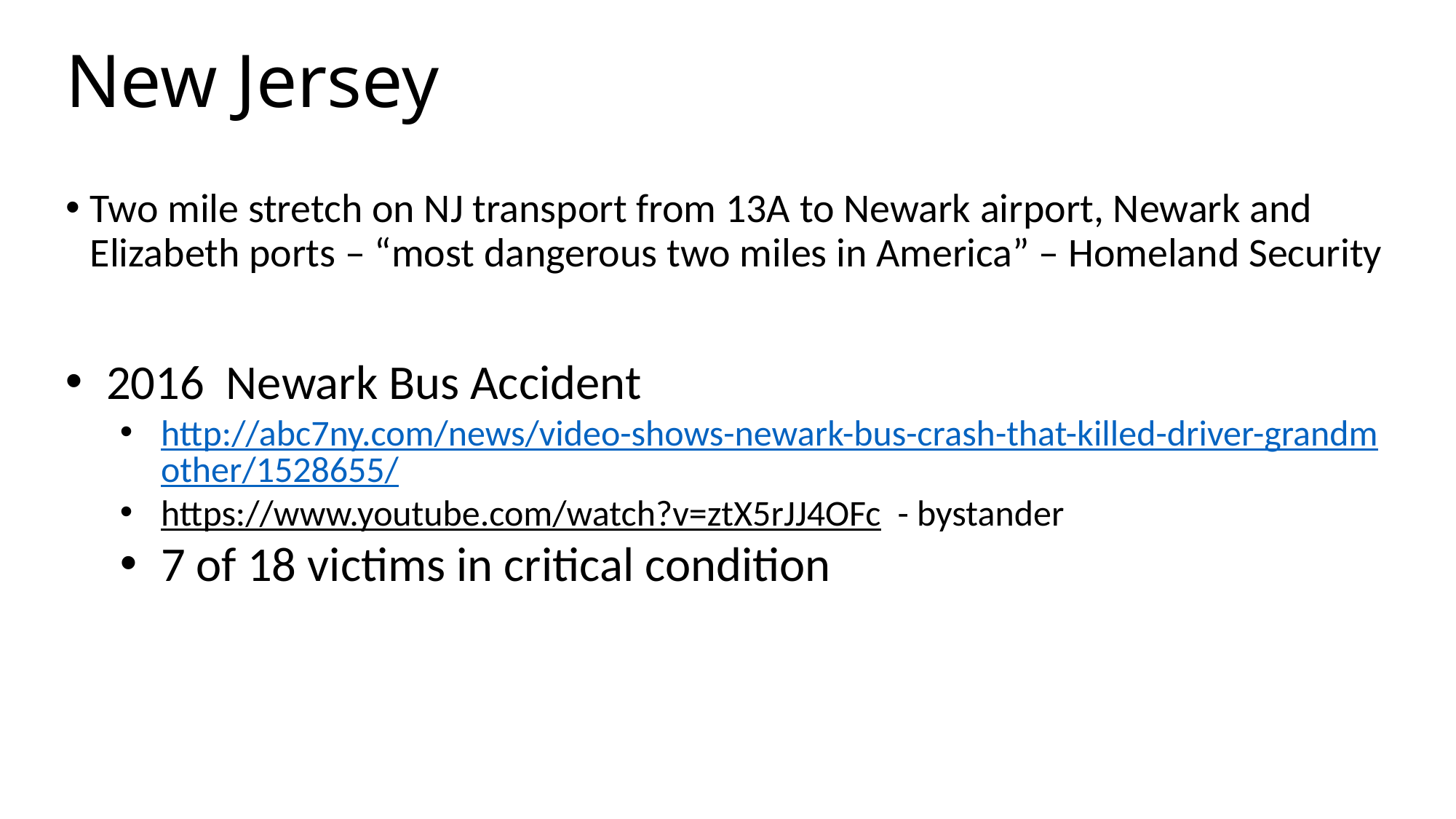

# New Jersey
Two mile stretch on NJ transport from 13A to Newark airport, Newark and Elizabeth ports – “most dangerous two miles in America” – Homeland Security
2016 Newark Bus Accident
http://abc7ny.com/news/video-shows-newark-bus-crash-that-killed-driver-grandmother/1528655/
https://www.youtube.com/watch?v=ztX5rJJ4OFc - bystander
7 of 18 victims in critical condition

## Slide 25
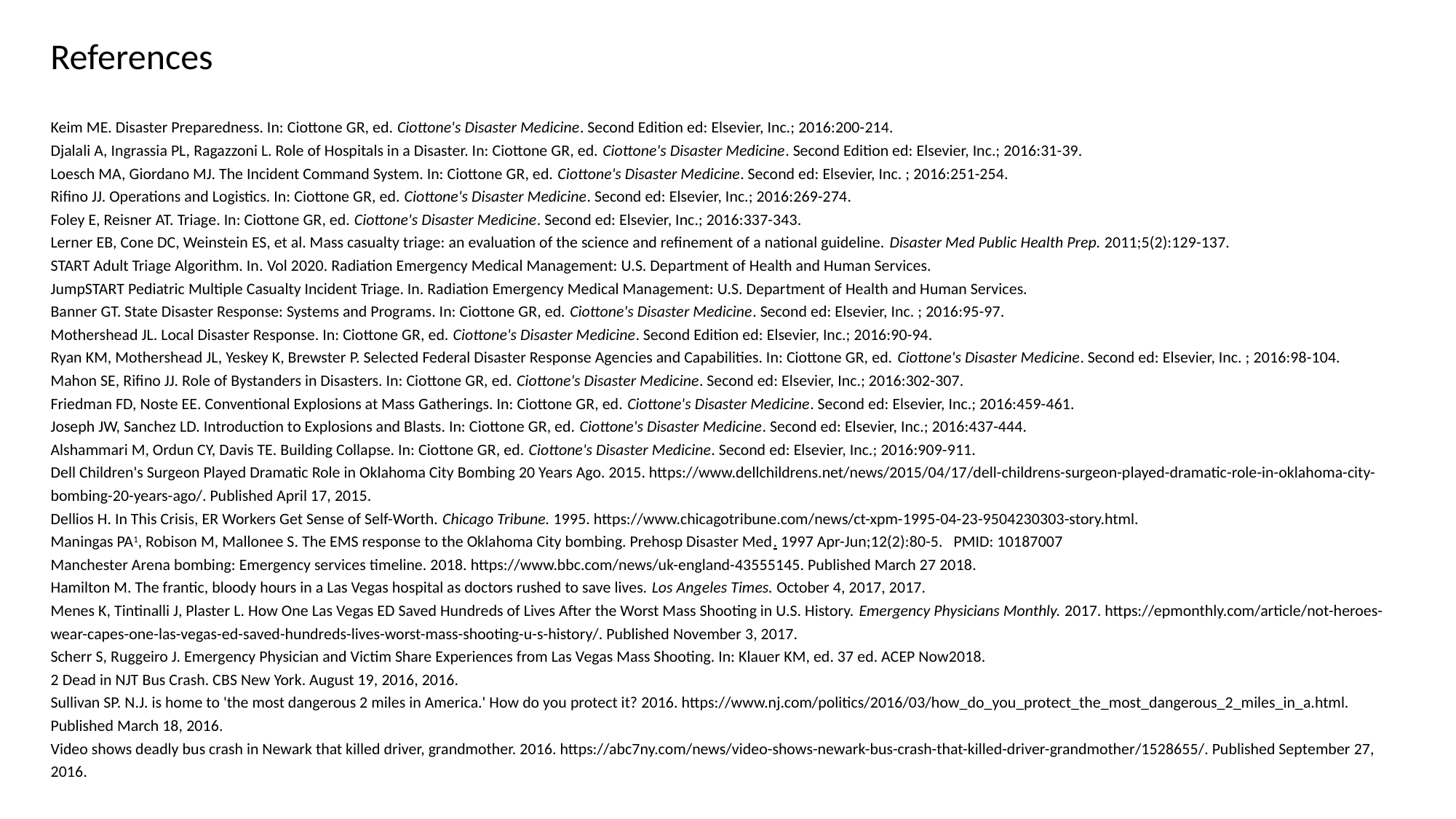

References
Keim ME. Disaster Preparedness. In: Ciottone GR, ed. Ciottone's Disaster Medicine. Second Edition ed: Elsevier, Inc.; 2016:200-214.
Djalali A, Ingrassia PL, Ragazzoni L. Role of Hospitals in a Disaster. In: Ciottone GR, ed. Ciottone's Disaster Medicine. Second Edition ed: Elsevier, Inc.; 2016:31-39.
Loesch MA, Giordano MJ. The Incident Command System. In: Ciottone GR, ed. Ciottone's Disaster Medicine. Second ed: Elsevier, Inc. ; 2016:251-254.
Rifino JJ. Operations and Logistics. In: Ciottone GR, ed. Ciottone's Disaster Medicine. Second ed: Elsevier, Inc.; 2016:269-274.
Foley E, Reisner AT. Triage. In: Ciottone GR, ed. Ciottone's Disaster Medicine. Second ed: Elsevier, Inc.; 2016:337-343.
Lerner EB, Cone DC, Weinstein ES, et al. Mass casualty triage: an evaluation of the science and refinement of a national guideline. Disaster Med Public Health Prep. 2011;5(2):129-137.
START Adult Triage Algorithm. In. Vol 2020. Radiation Emergency Medical Management: U.S. Department of Health and Human Services.
JumpSTART Pediatric Multiple Casualty Incident Triage. In. Radiation Emergency Medical Management: U.S. Department of Health and Human Services.
Banner GT. State Disaster Response: Systems and Programs. In: Ciottone GR, ed. Ciottone's Disaster Medicine. Second ed: Elsevier, Inc. ; 2016:95-97.
Mothershead JL. Local Disaster Response. In: Ciottone GR, ed. Ciottone's Disaster Medicine. Second Edition ed: Elsevier, Inc.; 2016:90-94.
Ryan KM, Mothershead JL, Yeskey K, Brewster P. Selected Federal Disaster Response Agencies and Capabilities. In: Ciottone GR, ed. Ciottone's Disaster Medicine. Second ed: Elsevier, Inc. ; 2016:98-104.
Mahon SE, Rifino JJ. Role of Bystanders in Disasters. In: Ciottone GR, ed. Ciottone's Disaster Medicine. Second ed: Elsevier, Inc.; 2016:302-307.
Friedman FD, Noste EE. Conventional Explosions at Mass Gatherings. In: Ciottone GR, ed. Ciottone's Disaster Medicine. Second ed: Elsevier, Inc.; 2016:459-461.
Joseph JW, Sanchez LD. Introduction to Explosions and Blasts. In: Ciottone GR, ed. Ciottone's Disaster Medicine. Second ed: Elsevier, Inc.; 2016:437-444.
Alshammari M, Ordun CY, Davis TE. Building Collapse. In: Ciottone GR, ed. Ciottone's Disaster Medicine. Second ed: Elsevier, Inc.; 2016:909-911.
Dell Children's Surgeon Played Dramatic Role in Oklahoma City Bombing 20 Years Ago. 2015. https://www.dellchildrens.net/news/2015/04/17/dell-childrens-surgeon-played-dramatic-role-in-oklahoma-city-bombing-20-years-ago/. Published April 17, 2015.
Dellios H. In This Crisis, ER Workers Get Sense of Self-Worth. Chicago Tribune. 1995. https://www.chicagotribune.com/news/ct-xpm-1995-04-23-9504230303-story.html.
Maningas PA1, Robison M, Mallonee S. The EMS response to the Oklahoma City bombing. Prehosp Disaster Med. 1997 Apr-Jun;12(2):80-5. PMID: 10187007
Manchester Arena bombing: Emergency services timeline. 2018. https://www.bbc.com/news/uk-england-43555145. Published March 27 2018.
Hamilton M. The frantic, bloody hours in a Las Vegas hospital as doctors rushed to save lives. Los Angeles Times. October 4, 2017, 2017.
Menes K, Tintinalli J, Plaster L. How One Las Vegas ED Saved Hundreds of Lives After the Worst Mass Shooting in U.S. History. Emergency Physicians Monthly. 2017. https://epmonthly.com/article/not-heroes-wear-capes-one-las-vegas-ed-saved-hundreds-lives-worst-mass-shooting-u-s-history/. Published November 3, 2017.
Scherr S, Ruggeiro J. Emergency Physician and Victim Share Experiences from Las Vegas Mass Shooting. In: Klauer KM, ed. 37 ed. ACEP Now2018.
2 Dead in NJT Bus Crash. CBS New York. August 19, 2016, 2016.
Sullivan SP. N.J. is home to 'the most dangerous 2 miles in America.' How do you protect it? 2016. https://www.nj.com/politics/2016/03/how_do_you_protect_the_most_dangerous_2_miles_in_a.html. Published March 18, 2016.
Video shows deadly bus crash in Newark that killed driver, grandmother. 2016. https://abc7ny.com/news/video-shows-newark-bus-crash-that-killed-driver-grandmother/1528655/. Published September 27, 2016.
