# Supplementary material for: Disaster Preparedness Training for Emergency Medicine Residents Using a Tabletop Exercise
Source: MedEdPORTAL. 2021 Mar 12;17:11119. doi: 10.15766/mep_2374-8265.11119 (PMC7970644; doi:10.15766/mep_2374-8265.11119)
Supplement: Supplementary file 1 — Exercise Lecture.pptxDisaster Scene Packet.docxHospital Scene Packet.docxPre-Exercise Survey.docxPostexercise Survey.docx [file mep_2374-8265.11119-s001.zip › B. Disaster Scene Packet.docx]

**Tabletop Exercise:**

**Disaster Scene Packet**

Included Sections:

1. Facilitator Guide

2. Example Timeline

3. Scene Map

4. Resources List

5. Triage Patients

6. Participant Instructions

**Tabletop Exercise – Facilitator Guide**

**Disaster Scene: Sheet 1**

*The italicized blue text on this page consists of instructions for facilitators to aid the participants during the drill. Do not read any of the facilitator instructions aloud to the participants. Un-italicized text is given to the participants on their corresponding sheets. The associated timeline instructs you when to hand each sheet to the group.*

*We placed the “victims” (indicated on index cards) in the corner of the room and taped the floor indicating a “warm zone” and a “hot zone”. Do not allow participants into this area until the prompt instructs you to allow them to do so.*

*Place 1 ALS (Advanced Life Support) truck and 2 BLS (Basic Life Support) trucks on the map ahead of time.*

Scenario:

It is Sunday, May 6^th^ at 8:30 pm. There is word that there was an explosion at the large indoor arena downtown during a major sporting event where there are reportedly about 20,000 people in attendance. It is a warm spring evening with temperatures around 60 degrees.

You are the Community Emergency Response Team (CERT) set to action.

You will find a map in front of you with information regarding the location of the explosion and nearby structures.

*Map contents: arena, idea of location of the explosion (on the north side of the building), nearby parking structures*

On scene: 1 Advanced Life Support (ALS) truck, 2 Basic Life Support (BLS) trucks

Tasks:

1. Give orders to units to begin responding to the scene and assessing the situation.
2. Establish roles amongst members according to the ICS (Incident Commander, Supporting Roles)
3. Designate triage, treatment, transport, staging, casualty collection/morgue areas

(*hand participants papers labeled with separate areas – triage, green treatment, yellow treatment, red treatment, black – they can physically place the patients in these areas later)*

1. You anticipate large masses of people. Where can you send patients and what do you have to consider when sending people there?

*Take into consideration the importance to not overburden hospitals – sending all the critical patients to one hospital, remembering the number of walking wounded that will potentially go to these hospitals and the number of patients they may already have.*

**Tabletop Exercise – Facilitator Guide**

**Disaster Scene: Sheet 2**

You have arrived on scene. Continue to set up your designated areas and incident command center. You note that people are filing out at rapid rates out of all emergency exits as firefighters attempt to enter the building and assess the scene.

Where will you direct these people to go?

*Decide on the map area to lead people away from the building safely, where to gather.*

Should you allow your EMS personnel to begin entering the building? What factors do you have to consider?

*Firefighters must go in first and ensure scene safety – no active fires, structural safety.*

*Consider that this detonation may be a terrorist attack – does it include biowarfare agents? Nuclear radiation? Could there be a second attack once first responders enter? Or a second, larger attack on the street?*

How will you protect the safety of the first responders?

*Stress the importance of PPE.*

*Learns should consider coordinating with police, homeland security and other potential law enforcement agencies about ensuring scene safety outside the building and closing down streets to use as space for patients.*

**Tabletop Exercise – Facilitator Guide**

**Disaster Scene: Sheet 3**

The fire department communicates that it appears this was a high order explosive with partial collapse of the stadium inside. They are there to provide assistance in extrication of patients and to ensure a safe zone for EMS. EMS are now permitted to enter the building.

Tasks: Send your crew to begin retrieving patients.

*Only allow participants to retrieve patients from designated warm zone. Do not allow them to simply pick up all the cards at once. They should simulate the ability to retrieve patients carrying out only one patient at a time. Encourage participants to triage patients in designated triage areas and not on scene. Participants should have some individuals designated to retrieve patients, others to triage and others to treat. Emphasize the importance of splitting up and designating roles.*

*As participants treat patients, an equipment card placed on the patient indicates that it has been used. Encourage them to physically keep patients in designated areas, so they may feel overwhelmed with the number of patients in the designated space and will want send patients to nearby hospital.*

*20 patients are in the warm zone.*

5 individuals arrive and inform you they are physicians and nurses and are here to help. How do you direct them?

*Direct them to staging area for assignments.*

*20 patients will be in the warm zone:*

*3 black, 6 red, 9 yellow, 2 green*

**Tabletop Exercise – Facilitator Guide**

**Disaster Scene: Sheet 4**

The fire department informs you they are finally able to reach some of the patients in the hot zone. Ambulances and crew from other towns begin to arrive.

*Allow participants access to the hot zone. Give them the rest of the ambulances and EMTs.*

*As they bring in the patients from the hot zone, start to distribute the cards for the patients that have updated vitals or change in clinical picture. This is done by switching the patient cards. Match the patients according to their identifying patient number. Not all patients will have changes. (See the list of patient descriptions.) This will only be done for the patients who have not yet been sent to a nearby hospital.*

One of your patients that was triaged as red calls out crying that a nearby victim has stopped talking to him. This prompts you to go back and reassess the patients you have triaged already.

*This prompt will encourage participants to reassess their patients.*

*4 total yellow patients become red.*

*1 red patient becomes black.*

*If they are sending too many patients to one hospital, you can prompt them by stating that there is a long line of ambulances at particular hospital.*

**Tabletop Exercise – Example Timeline**

**Disaster Scene**

***(For Facilitator ONLY)***

***8:00am***

- *Intro Lecture – 20 min*

***8:20am***

- **Place the following on the map ahead of participants arrival:*
  - *1 ALS Truck: 2 paramedics*
  - *1 BLS Truck: 2 EMTs*
  - *1 Police Car: 2 police officers*
- *Present group with map and “Sheet 1”*
- *Groups will assign roles and areas*

***8:30am***

- *Present the group with “Sheet 2”*

***8:35am***

- *Present the group with “Sheet 3”*
  - *If the group decides to use an ambulance to transport a patient to a local hospital, take the ambulance away from them for 2-3 min, before giving it back to them for use.*
  - *Give the group more ambulances at this time:*
    - *1 ALS*
    - *2 BLS*
    - *1 tactical rescue truck (3 EMTs)*
    - *1 EMS supervisor*
- *Allow the group access to warm zone only (You can say, “The fire department cleared this area for safety!”) 🡪 20 patients*

*NOTE:*

- *If the group is sending too many patients to one hospital, prompt the group with “The EMS Supervisor reports that the ambulance line is long at this hospital.”*
- *When they designate that a specific patient should go a certain hospital, place patient cards in each hospital area.*

***~8:45am (when they have a few warm zone patients left)***

- *Prompt the group with “Sheet 4” – ideally read this prompt to them rather than giving them the sheet.*
- *Allow the group access to the “hot zone” 🡪 10 patients*

***~8:50am***

- *Update the patients with their “reassessment descriptions”. (Just place the cards on top of patients if already triaged. If participants do not recognize that patients need to be re-triaged, prompt them.)*

***9:05am***

- *Give the group a 5-minute warning.*

***9:10am***

- *End the exercise and instruct group to reconvene for debrief.*

***9:15am***

- *Debrief*

***9:30am***

- *Concluding lecture*

***9:50am***

- *Finish*

**Tabletop Exercise – Scene Map**

**Disaster Scene**

*Instructions: For the disaster scene, the authors printed a map of the local downtown area including the location of the disaster and surrounding buildings, streets, and parking lots. For a scene scenario, readers could create or use a primitive map as shown, or customize a map to their local area. The purpose of the map is to allow participants to determine where different areas of the operations section would be located (where to triage, where to treat, stage, etc.)*


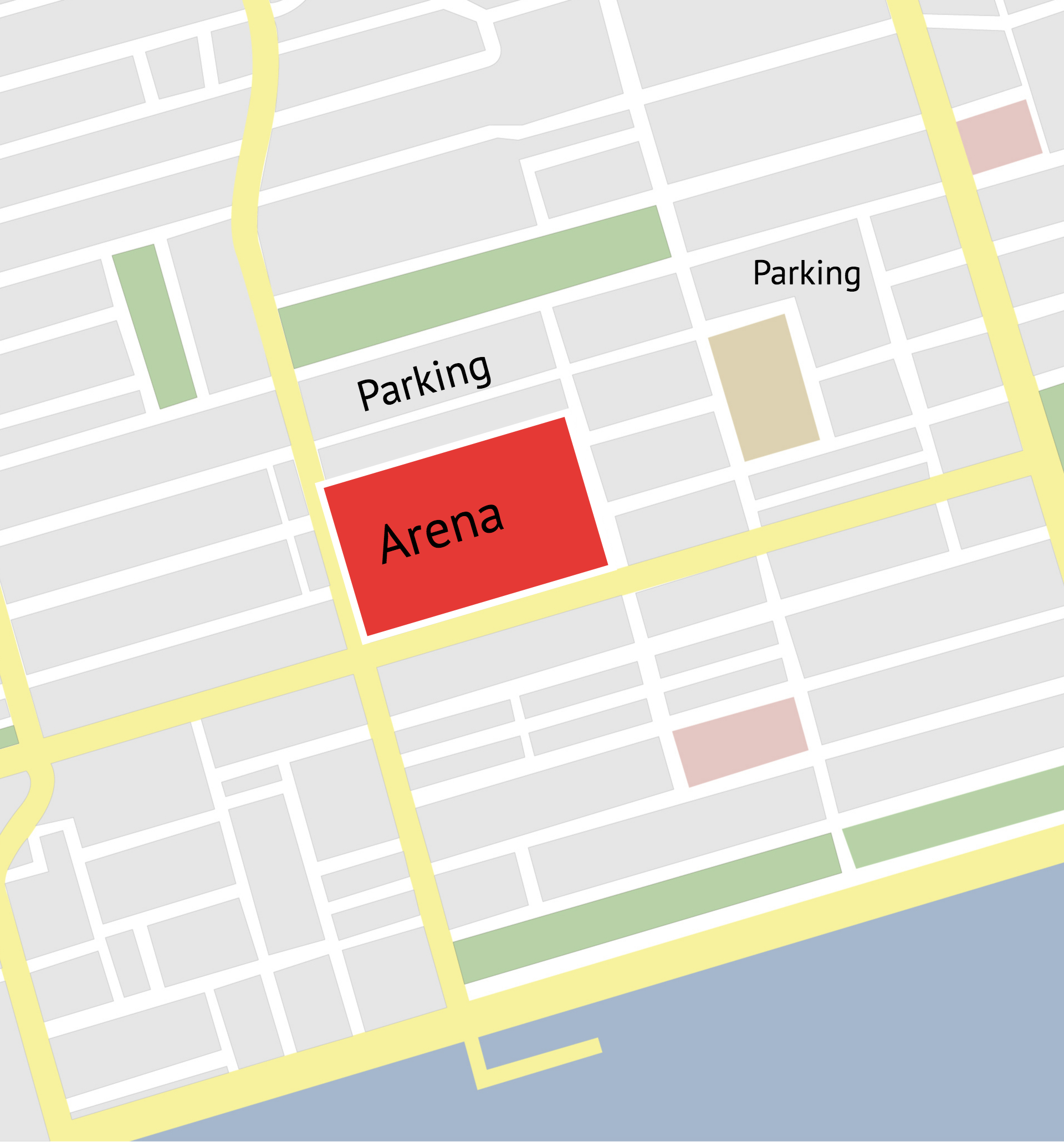


Author owned.

**Tabletop Exercise – Resources List**

**Disaster Scene**

Instructions: Print each of the items and paste on an index card. Each represents an available resource to participants at the disaster scene. Distribute to participants as instructed in facilitator guide. You can alternatively use pictures of the equipment available to your institution. It is also recommended that you use names of surrounding hospitals local to your institution.

| Ambulance | Ambulance | Ambulance |
| --- | --- | --- |
| Ambulance | Ambulance | Ambulance |
| Ambulance | Ambulance | EMT |
| EMT | EMT | EMT |
| EMT | EMT | EMT |
| EMT | EMT | EMT |
| Paramedic | Paramedic | Paramedic |
| Paramedic | Paramedic | Paramedic |
| Police Car | Police Car | Police Car |
| Rescue Helicopter | EMS Rescue Truck | Video Laryngoscope |
| Video Laryngoscope | Endotracheal Tube | Endotracheal Tube |
| Endotracheal Tube | Endotracheal Tube | Endotracheal Tube |
| Endotracheal Tube | Endotracheal Tube | Endotracheal Tube |
| Monitor | Monitor | Monitor |
| Monitor | Monitor | Monitor |
| Monitor | Monitor | Tourniquet |
| Tourniquet | Tourniquet | Tourniquet |
| Tourniquet | Tourniquet | Tourniquet |
| Tourniquet | Tourniquet | Tourniquet |
| Tourniquet | Tourniquet | Tourniquet |
| Tourniquet | Tourniquet | Tourniquet |
| Tourniquet | Tourniquet | Tourniquet |
| Tourniquet | Tourniquet | Tourniquet |
| Tourniquet | Tourniquet | Tourniquet |
| Tourniquet | Tourniquet | Tourniquet |
| Tourniquet | Tourniquet | Tourniquet |
| BVM | BVM | BVM |
| BVM | BVM | BVM |
| BVM | BVM | BVM |
| BVM | BVM | BVM |
| BVM | BVM | BVM |
| BVM | BVM | BVM |
| BVM | BVM | BVM |
| BVM | BVM | BVM |
| BVM | BVM | BVM |

Level 1 Trauma Center

Level 2 Trauma Center

Community Hospital A

Community Hospital B

**Tabletop Exercise – Triage Patients**

**Disaster Scene**

| Instructions:  Each patient indicated by a number. Cut out each patient description and paste on an index card. Do not include triage color on index card. Be sure to include the number. That will allow you to refer to this answer key to ensure that participants are completing proper tagging. Some patients have changes on reassessment. Cut these descriptions and place on separate index card. Numbers will allow you to place the correct reassessment description on top of the right patient. Do this when indicated by the facilitator guide and timeline. Patients under section “warm zone” are to be given to participants first, as instructed by facilitator guide. Give the patients under “hot zone” only after instructed to do so by facilitator guide. | | | |
| --- | --- | --- | --- |
| Initial Patient Encounter Description | Triage color | Reassessment Description | Triage color |
| WARM ZONE | | | |
| 1. Young adult female found with large laceration and severe palpable deformity of the skull. Her eyes do not open. She is not making any sounds, and her arms flex in response to painful stimuli. | Black |  |  |
| 2. Middle aged male found to be apneic. You reposition his airway, but there are no spontaneous respirations. | Black |  |  |
| 3. Middle aged female discovered to be spontaneous breathing. She is making incomprehensible sounds and does not open her eyes. She withdraws to painful stimuli. You note full thickness burns throughout bilateral lower and upper extremities (anterior and posterior), entire anterior trunk, genitals and lower posterior trunk. | Black |  |  |
| 4. Middle aged female with obvious trauma to head. She opens her eyes to painful stimuli, withdraws to pain and is speaking with inappropriate words. | Red | 4. She does not open her eyes, makes no verbal sounds and extends her upper extremities to painful stimuli. | Black |
| 5. Middle aged man with wound to his chest. He is spontaneous breathing at a rate of 32. He opens his eyes to voice, is confused and withdraws to pain. | Red | 5. He has become more tachypneic and confused. He opens his eyes to painful stimuli.  Vitals: HR 140, RR 38, BP 84/38, O2 85% | Red |
| 6. Young adult male with injuries to his L arm and R leg. There is active bleeding from both sites (the lower extremity has a partial amputation) as well as partial and full thickness burns to the anterior lower extremities and trunk. He does not answer questions but is breathing spontaneously, eyes are open and he follows gestures.  Vitals: HR 135, RR 28, BP 90/60, O2 95% | Red | 6. No change in his clinical assessment.  Repeat vitals:  Vitals: HR 138 RR 28 BP: 88/55 O2 95% | Red |
| 7. Young adult female with partial thickness burns to her posterior trunk and upper extremities. She has a piece of metal in her right lower quadrant. She is spontaneously breathing and follows commands.  Vitals: HR 132 RR 32 BP: 98/60 O2 96% | Red | 7. Repeat vitals:  Vitals: HR 138, RR 30, BP 86/59, O2 96% | Red |
| 8. Middle aged female with obvious head injury. She opens eyes to painful stimuli, is confused and localizes to pain. She has a closed deformity of the right lower extremity and partial thickness burns of her left lower and upper extremities.  Vitals: HR 95, RR 32, BP 100/68, O2 94% | Red | 8. She opens eyes to painful stimuli, answers with inappropriate words and withdraws to pain.  Vitals: HR 60, RR 14, BP 88/58, O2 90% | Red |
| 9. Young adult male with partial amputated left lower extremity. His eyes are open, and he is yelling in pain and speaks in clear words. There is active bleeding noted from the amputation site.  His capillary refill time is less than 2 seconds.  Vitals: HR 120, RR 28, BP 95/70, O2 100% | Red | 9. Vitals:  HR 110, RR 25, BP 98/72, O2 100% | Red |
| 10. Middle aged female with trauma to the head. She is spontaneously breathing at a rate of 28. She has palpable radial pulses and is able to obey commands. She complains of some abdominal pain. She rests with her eyes closed but opens them to voice.    Vitals: HR 105, RR 24, BP 96/58, O2 100% | Yellow | 10. She is now diaphoretic, opens eyes to painful stimuli, and mumbles incomprehensible sounds. She withdraws from pain.  Vitals: HR 128, RR 32, BP 87/59, O2 90% | Red |
| 11. Young adult male with an open fracture of the right lower extermity and closed deformity of the RUE. There are partial thickness burns of the right upper extremity and anterior right lower extremity. He is spontaneously breathing at a rate of 26, has palpable radial pulse on the left, and follows commands. He is oriented but complaining of severe pain.  Vitals: HR 112, RR 26, BP 100/70, O2 100% | Yellow | You find there is continued bleeding below the site of the tourniquet. He is now confused, opens eyes to voice, and localizes to pain.  Vitals: HR 130, RR 30, BP 88/60, O2 93% | Red |
| 12. Young adult female with full thickness burns to the anterior left upper extremity and partial thickness to the anterior left lower extremity. She complains of left upper quadrant pain and left back pain. She is able to follow commands and has palpable radial pulses. She is not able to ambulate.  Vitals: HR 108, RR 22, BP 98/58, O2 100% | Yellow | She is now diaphoretic and confused but repeatedly moaning in pain. She is moving her extremities spontaneously and opens her eyes to voice.  Vitals: HR 132, RR 32, BP 82/60, O2 92% | Red |
| 13. Teenage female with small laceration on her forehead. She is unable to move her legs.  RR 20, strong pulse | Yellow |  | Yellow |
| 14. Young adult female found with a piece of shrapnel in her R eye. Her radial pulse is present and she is following commands.  Vitals: HR 110, RR 28, BP 103/78, O2 100% | Yellow |  | Yellow |
| 15. Young gravid female with a left upper extremity deformity. She is breathing and follows commands. Her left radial pulse is absent, but her right radial pulse is present.  Vitals: HR 96, RR 27, BP 98/70, O2 100% | Yellow |  | Yellow |
| 16. Middle aged male breathing spontaneously with clear deformities of the right upper and lower extremities. The right lower extremity is open, but there is no noted external bleeding. There are also partial thickness burns of his left upper extremity and half of the upper chest.  Vitals: HR 110, RR 27, BP 105/80, O2 100% | Yellow |  | Yellow |
| 17. Middle aged female with a hematoma and abrasion on her forehead. She complains of lower back pain and is unable to move her legs. Her pulses are present and she is awake. | Yellow |  | Yellow |
| 18. Young adult male with partial thickness burns to the posterior lower extremities. | Yellow |  | Yellow |
| 19. School aged boy with partial thickness burns on his bilateral anterior upper extremities. He is ambulating without difficulty. He does not respond when you ask him questions but follows gestures. | Green |  | Green |
| 20. Young adult male who is walking but holding his arm with an obvious deformity. | Green |  | Green |
| HOT ZONE | | | |
| 21. A 2 yo child is pulled from rubble. He is not breathing and is unresponsive. | Black | 21. Rescue breaths given, remains apneic | Black |
| 22. A middle aged male is found with his legs trapped under rubble. There is a piece of wood impaled in the neck. He is unresponsive and breathing once every second. His capillary refill is 4 seconds. | Black |  |  |
| 23. A young adult female was also pulled from the rubble. She is breathing at a rate of 45 per minute. She complains of severe chest pain and difficulty breathing. She is awake and alert and her capillary refill is less than 2 seconds. | Red |  | Red |
| 24. A teenage boy is found with a metal rod impaled in his abdomen. He is awake and alert and breathing once every 4 seconds. He complains of breathing difficulties and cannot remember what happened. His capillary refill is greater than 4 seconds. | Red |  | Red |
| 25. A young adult male is found with blood spurting from a neck injury. He is breathing at a rate of 34 and is awake. He has a deformity of his left lower extremity. | Red |  | Red |
| 26. A middle aged male is found to be breathing rapidly. He has a laceration on his forehead. His chest is sinking in on respirations. His radial pulses are palpable. | Red |  | Red |
| 27. A middle aged female is extricated from beneath a partially collapsed wall. Her eyes are closed and she does not respond to questions. She is breathing once every second. Her capillary refill is less than 2 seconds. | Red |  | Red |
| 28. A young adult female is recovered from rubble with a prolonged extrication time. She is awake and has a palpable pulse. She complains of abdominal pain and her right upper extremity has an open deformity. | Yellow | 28. She becomes confused and unable to follow commands. Her radial pulses are weak and thready. | Red |
| 29. A teenage boy is discovered with gross deformities to both feet that were crushed with a concrete block. He is breathing once every 3 seconds and can recall what happened. His capillary refill is less than one second. | Yellow |  | Yellow |
| 30. A young adult male is found with burns to the bilateral anterior lower extremities with a large laceration on the thigh that is actively bleeding. He is alert and awake and breathing at a rate of 28 breaths per minute. His radial pulses are palpable bilaterally. | Yellow |  | Yellow |

**Tabletop Exercise**

**Disaster Scene: Sheet 1**

Scenario:

It is Sunday, May 6^th^ at 8:30 pm. There is word that there was an explosion at the large indoor arena downtown during a major sporting event where there are reportedly about 20,000 people in attendance. It is a warm spring evening with temperatures around 60 degrees.

You are the Community Emergency Response Team (CERT) set to action.

You will find a map in front of you with information regarding the location of the explosion and nearby structures.

On scene: 1 Advanced Life Support (ALS) truck, 2 Basic Life Support (BLS) trucks

Tasks:

1. Give orders to units to begin responding to the scene and assessing the situation.
2. Establish roles amongst members according to the ICS (Incident Commander, Supporting Roles)
3. Designate triage, treatment, transport, staging, casualty collection/morgue areas
4. You anticipate large masses of people. Where can you send patients and what do you have to consider when sending people there?

**Tabletop Exercise**

**Disaster Scene: Sheet 2**

You have arrived on scene. Continue to set up your designated areas and incident command center. You note that people are filing out at rapid rates out of all emergency exits as firefighters attempt to enter the building and assess the scene.

Where will you direct these people to go?

Should you allow your EMS personnel to begin entering the building? What factors do you have to consider?

How will you protect the safety of the first responders?

**Tabletop Exercise**

**Disaster Scene: Sheet 3**

The fire department communicates that it appears this was a high order explosive with partial collapse of the stadium inside. They are there to provide assistance in extrication of patients and to ensure a safe zone for EMS. EMS are now permitted to enter the building.

Tasks:

Send your crew to begin retrieving patients.

5 individuals arrive and inform you they are physicians and nurses and are here to help. How do you direct them?

**Tabletop Exercise**

**Disaster Scene: Sheet 4**

The fire department informs you they are finally able to reach some of the patients in the hot zone. Ambulances and crew from other towns begin to arrive.

One of your patients that was triaged as red calls out crying that a nearby victim has stopped talking to him. This prompts you to go back and reassess the patients you have triaged already.
