# Supplementary material for: Disaster Preparedness Training for Emergency Medicine Residents Using a Tabletop Exercise
Source: MedEdPORTAL. 2021 Mar 12;17:11119. doi: 10.15766/mep_2374-8265.11119 (PMC7970644; doi:10.15766/mep_2374-8265.11119)
Supplement: Supplementary file 1 — Exercise Lecture.pptxDisaster Scene Packet.docxHospital Scene Packet.docxPre-Exercise Survey.docxPostexercise Survey.docx [file mep_2374-8265.11119-s001.zip › C. Hospital Scene Packet.docx]

**Tabletop Exercise**

**Hospital Scene Packet**

Included Sections:

1. Facilitator Guide

2. Example Timeline

3. Scene Map

4. Sign-out List for Facilitator

5. Sign-out List for Participants

6. Patients to be Triaged in ED

7. Hospital Resources List

8. Participant Instructions

**Tabletop Exercise – Facilitator Guide
Hospital Scene: Sheet 1**

*Instructions for facilitators are listed in italicized blue font. Do not read any of the facilitator instructions aloud to the participants. The “correct answers” to many of the prompts reflect our hospital’s actual disaster plan. You may alter the exercise to reflect your own institution’s actual plan.*

*Present the following scenario to the group.*

Scenario:

It is Friday, February 24^th^ at 9:45PM. EMS informs you that there is an apartment fire nearby in a large high-rise building, and you should anticipate a large number of patients. You do not know how many could be coming at this point. The scene is still being processed, and the number of patients and their acuity is unknown. You must quickly prepare for a large influx of patients from the scene.

Staff Currently Present in ED:

*The following reflects our regular staffing. You can adjust to reflect your own staffing in your emergency department.*

| **Staff Qualification** | **Number Present** | **Staff Qualification** | **Number present** |
| --- | --- | --- | --- |
| EM Attendings: | 3 Adult attendings | Nurse Practitioners | 2 |
|  | 1 Ped attending | Nurses | 23 |
| Trauma Surgeons: | 1^st^ - in Hospital | Nurse Techs | 7 |
|  | 2^nd^ - Home Call |  |  |
| EM Residents: |  |  |  |
| EM4 | 1 | 4^th^-year Medical Students | 1 |
| EM3 | 2 | IM Residents (PGY-2) | 2 |
| EM2 | 2 |  |  |
| EM1 | 3 |  |  |

You will find maps of the emergency department in front of you with the current patients in each section of the department.

**Tasks**:

1. Prepare for arrival of patients from the scene.
2. As the emergency department, you are part of the operations section. Assign roles amongst your staff such as who will be the attending physician, resident physicians, charge nurse, etc. What specific jobs will each person be doing when patients arrive?

*Participants should divide themselves into section leader, triage leader, treatment leader, staging leader. Additionally, they should assign individuals to different treatment areas (red, yellow, etc.).*

1. Where will EMS bring patients?

*The team should recognize that the most efficient space for triage is likely not where our daily triage takes place. Consider designating an area outside, redirecting the flow of traffic of the ambulances, not allowing police cars to park in the ambulance bay as they usually do, etc. Enlist security to help keep the flow of traffic and driveways clear.*

1. Where are you going to put patients?

*Team should designate areas to set up triage, different treatment areas (where will all the red tagged patients go? The yellow, green, etc.)*

1. How are you going to decide who will care for who?

*They should reconsider the staffing of residents, medical students, etc. and consider rearranging staffing models to staff each area and have clear roles.*

1. How will you continue to care for the patients already in the emergency department?

*Encourage them to get the admitted patients upstairs. Try to get patients with ALMOST complete workups upstairs.
Get all the discharged patients out of the department.
They can consider rearranging the space. The curtains between rooms can be opened to fit more than one patient per room, etc.*

***Evaluate how the group did the following:***

1. ***Did the group assign roles?***

*Senior ED Physician = Treatment Unit Leader Officer*

*Charge Nurse = ED Branch Director*

*Physician in charge of triage, treatment, etc.*

1. ***Get as many of the current patients out of the ED as possible***

- *Get discharged patients out of ED*
- *All admitted patients upstairs*
- *All pending admissions admitted*
- *Move out all patients who do not need monitored beds to other areas – dental clinic, outpatient clinics, other clinic areas*

1. ***Did the team utilize other spaces?***

- *The Cancer Center and clinic on campus can be used to see patients.*
- *Triage patients in the parking lot/ambulance bay*

1. ***How did the team redistribute/assign staff?***

- *Physicians should have designated areas – treatment unit leader, triage unit leader, etc.*
- *Internal medicine (IM) or rotating residents from other specialties could see and triage patients in the waiting room*
- *Acute care nurses from other units can be called to help in ED.*
- *Other physicians who could help should report to ED – trauma, general surgery, critical care, PICU, etc.*
- *Other staff to report to the ED: Radiology supervisor, pharmacy, charge RN, blood bank supervisor, hospital transport bring all stretchers and wheelchairs to ED, respiratory therapists, portable XR and technician, etc.*

**Tabletop Exercise – Facilitator Guide**

**Hospital Scene: Sheet 2**

Patients begin arriving to the emergency department. You are told that there was a building collapse and patients are starting to be pulled from the rubble. Simultaneously, many physicians and nurses begin to arrive to the emergency department from home and begin to clog the main ED and trauma bay. Also, members of the public arrive looking for family members they are worried were in the collapse.

Does your current plan work for the incoming patients? What adjustments can be made?

What are you going to do with the extra staff? How will you verify their credentials?

*All extra staff (those not specifically assigned to the ED are to report to the designated staging area for validation and assignment. (In our hospital, this is the cafeteria. Participants may choose another location for this to take place.)*

How are you going to rapidly register the large volume of patients?

**Specific to our hospital: There is a mass casualty incident packet and disaster box containing radios, vests to identify leaders and roles, Department of Health triage tags with medical record numbers and green bands to label blood products. This quickly identifies and “registers” patients that can be merged with electronic charts at a later time.*

How will you handle the family members?

**Specific to our hospital: Family members are to be directed to the nearby dental school where patient representatives and family services will assist the families. Participants should consider where family members can gather and who will provide them with information and address their concerns.*

*Ask the team about security if they did not discuss it. Where should security form a perimeter? Security becomes more aggressive during a disaster and all visitors are asked to leave.*

*Start handing out the patients slowly or in a non-rhythmic pattern in attempt to reflect actual arrival of patients from a disaster scene.*

*After about half of the mass casualty victims have presented to the ED, include the presentation of the stroke patient. This simulates that despite a mass casualty incident that may be currently afflicting a hospital, other patients with critical ailments may continue to arrive and should also receive appropriate care.*

**Tabletop Exercise – Example Timeline**

**Hospital Scene
(For Facilitator ONLY)**

**8:00am**

- Intro Lecture – 20 min

**8:20am**

- Present the group with the map and “Sheet 1” of instructions
  - Participants should assign roles and designate areas on map.
- Allow the group time to get to know their current patients – provide participants with the “sign out” list.

**8:30am**

- Prompt group that a demented patient falls out of bed. (See ED list of patients).

**8:40am**

- Present group with “Sheet 2”.
- Start handing out notecards representing patients from the scene: start with green patients, then yellow and red.
- Ensure the participants are “registering” patients in some manner. (Each group may do this differently.)
- Prompt the group that large group of family members has arrived.
- Prompt the group that large groups of volunteer healthcare workers are arriving in the ED asking to help, but clogging the hallways.

**8:45am**

- Prompt the group that the asthma patient tries to elope and collapses. (See list of patients.)

**8:47**

- Prompt the group that the suicidal patient eloped.

**8:50**

- Present the group with the patient card representing the incoming patient with a stroke.

**8:55**

- Prompt the group that the overdose patient was found cyanotic and not breathing in bathroom.
- If group finishes with extra time, allow a small group debrief.

**9:10am**

- 5 min warning

**9:15am**

- Debrief

**9:30am**

- Closing Lecture

**9:50am**

- End

**Tabletop Exercise – Scene Map**

**Hospital Scene**


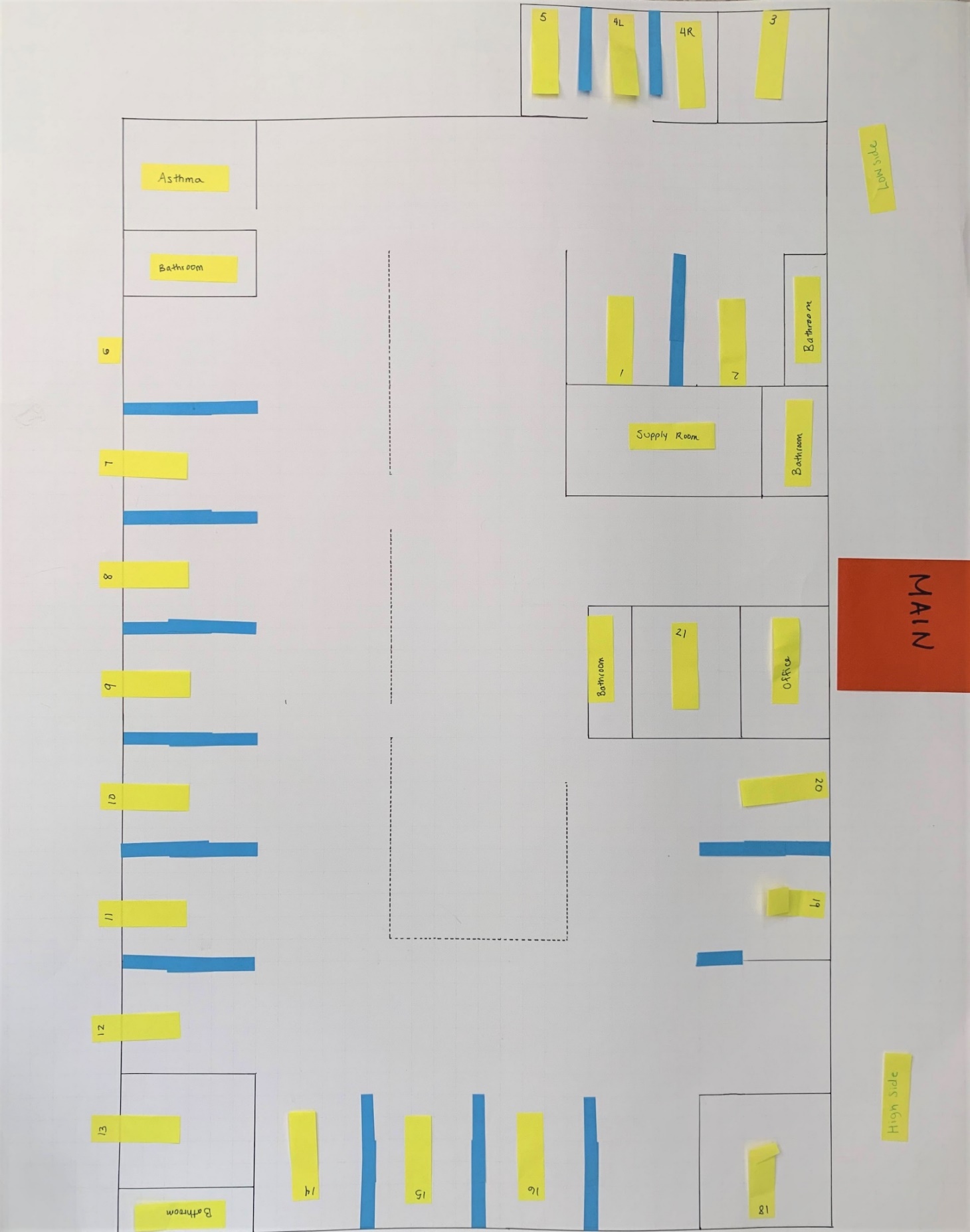


Author owned.

Instructions: This is a photo of a map of our main emergency department drawn by the authors on graph poster paper. We created similar maps for different sections of the department (e.g. pediatrics, trauma, etc.). Blue sticky-notes were used to indicate curtains. This was done to allow participants to remove the curtains if they choose and rearrange the department as needed. Yellow sticky-notes simply indicate room numbers. It is encouraged that you create a similar map of your own department.

**Tabletop Exercise**

**Hospital Scene**

**Sign Out List for Facilitator**

*This list is similar to our hospital’s ED track board. Our participants recognize the meaning of bed and status without us having to provide the key to them. This list could be altered to reflect track boards in individual emergency departments.*

**Key:**
Bed: A = Main Adult ED; AH = Main Hallway; AS = main asthma chair; C = crisis (psych ED); M = Midtrack area; P = pediatric ED, FT = fast track; FTC = Fast track chair; WR = waiting room
Status: A = admitted; P = pending disposition; DC = Discharged; P:obs = pending acceptance to observation unit; MC = medically cleared for psychiatric eval; TBS = To be seen;

CC = chief complaint; WNL = within normal limits; Vitals are presented in following order: Heart Rate, Blood Pressure, Respiratory Rate, Oxygen Saturation, Temperature (in Fahrenheit); FS = point of care glucose measurement; NC = nasal cannula, NRB = non-rebreather, BIPAP = bilevel positive airway pressure;

| **Bed** | **Status** | **Pt** | **CC** | **Vitals** | **Sign Out Notes** | **Developments in Course** | **Notes For Facilitator** |
| --- | --- | --- | --- | --- | --- | --- | --- |
| A1 | A | 63 yo M | Dyspnea | 110, 120/82, 18, 92%, 98.8, FS 98 | CHF exacerbation. Receiving dialysis in emergency department. |  |  |
| A2 | TBS | 76 yo F | Chest Pain | 98, 156/87, 18, 100%, 98.7 | Complaining of chest pain for 2 hours. History of MI. |  |  |
| A3 | A | 73 yo F | Cough, Fever | 93, 164/97, 22, 93% on NC, 100.3 | Rule out tuberculosis. |  |  |
| A4L | MC | 34 yo F | Behavioral Problem | WNL | Suicidal ideations |  |  |
| A4R | MC | 38 yo M | Alcohol Problem | WNL | Was intoxicated, now sober; Complaining of suicidal ideations |  |  |
| A5 | MC | 49 yo M | Behavioral Problem | WNL | Aggressive with family at home, history of schizophrenia |  |  |
| A6 | DC | 30 M | Abdominal Pain | WNL | Presented with nausea/vomiting/abdominal pain; CT abdomen/pelvis negative; Tolerated PO |  |  |
| A7 | P | 54 yo M | Chest Pain | 88, 172/95, 18, 100%, 98.7 | Pending 2nd troponin (to be drawn in 1 hour) and ECG; HEART score of 2. Everything else negative. |  |  |
| A8 | P | 38 yo M | Asthma | 112, 139/94, 24, 98% on BIPAP, 99.3 | Currently on BIPAP; Wants to leave against medical advice. | Tries to leave elope and then collapses in hallway. More tachypneic, and increased work of breathing. | Pt is responsive. If group places patient back on BIPAP, patient improves. |
| A9 | A | 82 yo M | Cough | 110, 158/87, 26, 91% on NC, 100.5 | Admitted to step down unit for 2 days. |  | Should see if can be downgraded floor bed and get patient upstairs. |
| A10 | P | 63 yo M | Seizures | 90, 105/86, 12, 100% on ventilator, T99.4 | Presented with status epilepticus for 20 minutes, intubated for airway protection; on propofol and fentanyl drips; Pending labs and head CT |  |  |
| A11 | P | 33 yo F | Fever, Chills  Sore throat | 103, 129/84, 14, 100%, 101.4 | Exam: No muffled voice, difficulty breathing; Tolerating secretions; Large edema of the right peritonsillar space, erythematous, no plaques on tonsils Labs unremarkable. Peritonsillar abscess found on bedside ultrasound. Pending drainage. |  | Should offload this procedure (i.e. call ENT, or send to obs for ENT to do it later)  Move this patient for procedure, maybe to dental clinic? Fast track? |
| A12 | A | 67 yo F | Abdominal Pain | 102, 148/94, 18, 99%, 100.6 | Admitted to surgery for acute cholecystitis. Receiving antibiotics. |  |  |
| A13 |  |  |  |  |  |  |  |
| A14 | P | 58 yo M | Dyspnea | 127, 188/105, 30, 94% on BIPAP, 99.1 | Presented 10 minutes ago in respiratory distress with diffuse rales and hypertensive to 200/115. Immediately placed on BIPAP and started nitroglycerin drip. Pt appears mildly more comfortable at this time.  ECG: T wave inversions (no prior for comparison) BIPAP settings: 10/5 60% RR12 | Repeat Vitals: 130, 170/100, 32, 85% Pt looks really uncomfortable, working very hard to breathe. | Group should note that BIPAP settings need to be adjusted. Pt will stabilize after this. |
| A15 | A | 38 yo M | Abdominal Pain | 99, 110/80, 26, 98%, 100.2,  FS 540 mg/dL | Admitted to ICU for severe DKA, on insulin drip with bicarb infusion |  | Participants may consider checking repeat point of care blood glucose. |
| A16 | DC | 56 yo M | Edema | WNL | Diagnosed with cellulitis, plan to discharged home with oral antibiotics. Labs/imaging unremarkable. |  |  |
| A17 | P | 86 yo M | AMS | 92, 156/97, 18, 98%, 99.3 | History of dementia and frequent falls; Brought in by family for worsening bizarre behavior, increasing forgetfulness.  History of A fib on warfarin. | Pt found on floor next to bed.  Repeat vitals: 60, 150/90, 10, 95%  FS 90 mg/dL  More altered and lethargic. | If group decides to get CT head, tell them there is a large subdural hematoma. Pt needs to be intubated. |
| A18 | P | 56 yo M | Blood Sugar Problem | 102, 148/92, 18, 99%, 98.6 FS 430 mg/dL | Noticed FS at home was high but otherwise asymptomatic. ECG normal, VBG normal, pending rest of labs. Receiving intravenous fluids. |  | Will just need fluids to bring down FS, no DKA, HHS or other reason to admit patient. |
| A19 | P | 63 yo M | Chest Pain | 78, 160/90, 18, 99%, 98.5  FS 103 mg/dL | History of diabetes, CAD, HTN who presented with chest pain for one day. ECG and CXR are normal, pending labs. |  |  |
| A20 | P | 48 yo F | Drug Problem | 107, 164/96, 10, 93% on NC, 99.5,  FS 90 md/dL | Brought in by EMS after opioid overdose. Received 0.4 mg IV naloxone in the field. Now breathing and drowsy. |  |  |
| A21 | A | 82 yo F | Breathing Problem | 110, 150/80, 20, 94%, 101.2 | Presented with shortness of breath and weakness. Recently treated for pneumonia in hospital. Now also complaining of multiple episodes of diarrhea; Admitted for rule out C diff. |  |  |
| AH1 | TBS | 38 yo M | Back Pain | 68, 130/80, 18, 98%, 98.8 | Back pain since heavy lifting yesterday |  |  |
| AH2 | P | 48 yo F | Alcohol Problem | WNL | One of our regular patients admits to ETOH use today. No signs of trauma. |  |  |
| AH3 | P | 47 yo F | Opioid Overdose | 70, 148/96, 12, 99%, 98.7  FS 109 mg/dL | Received naloxone in field by EMS. Sitting in bed awake and alert, denies using heroin or other drugs, wants to leave. | Pt found in bathroom cyanotic and not breathing with heroin on bathroom floor. Pulse is present. | Group will need to resuscitate patient - will only require respirations and naloxone. Pt will awake and stabilize. |
| AH4 | MC | 29 yo M | Behavioral Problem | WNL | Complaining of hallucinations. |  |  |
| AH5 | P | 23 yo F | Vaginal Bleed | 82, 102/80, 16, 99, 98.6 | 8 weeks pregnant with spotting for one day. Confirmed intrauterine pregnancy on ultrasound. Pending labs. |  |  |
| AH6 | P | 61 yo F | Suicidal Ideations | WNL | History of suicide attempt in past. Complains of suicidal ideations with plan. | Elopes |  |
| AH7 |  |  |  |  |  |  |  |
| AH8 | P: Obs | 29 yo F | Vomiting | WNL | Presented with vomiting and right lower quadrant pain for one day. Labs and CT negative. Pending transfer to observation for hydration and PO challenge. |  |  |
| AH9 | TBS | 33 yo M | Rash | 90, 110/80, 18, 98%, 98.9 | Triage note: Rash for 2 days |  |  |
| AH 10 |  |  |  |  |  |  |  |
| AH 11 |  |  |  |  |  |  |  |
| AH 12 |  |  |  |  |  |  |  |
| AS1 | P | 55 yo M | Breathing Problem | 110, 120/90, 22, 92%, 98.7 | One of our regular patients with wheezing on exam. |  |  |
| AS2 | P | 58 yo F | Breathing Problem | 105, 150/98, 18, 93%, 98.9 | One of our regular patients with wheezing on exam. |  |  |
| C1 | MC | 38 yo F | Behavioral Problem | WNL | Suicidal ideations |  |  |
| C2 | MC | 49 yo M | Behavioral Problem | WNL | Brought in by police for aggressive behavior in street. Received 5 mg haldol and 5 mg versed IM. |  |  |
| C3 | MC | 29 yo F | Behavioral Problem | WNL | Hallucinations |  |  |
| C4 | MC | 37 yo M | Behavioral Problem | WNL | Suicidal ideations |  |  |
| C5 |  |  |  |  |  |  |  |
| C6 | MC | 53 yo F | Behavioral Problem | WNL | Suicidal ideations |  |  |
| M1 | TBS | 47 yo F | Leg Swelling |  |  |  |  |
| M2 | TBS | 24 yo M | Leg Pain |  | Obvious deformity on exam. |  |  |
| M3 | TBS | 55 yo M | Back Pain |  |  |  |  |
| M4 | TBS | 32 yo F | Abdominal Pain |  |  |  |  |
| M5 |  |  |  |  |  |  |  |
| M6 |  |  |  |  |  |  |  |
| P1 |  |  |  |  |  |  |  |
| P2 | DC | 7 yo M | Fall | WNL |  |  |  |
| P3 | P | 19 yo F | Vaginal Bleeding | 90, 105/80, 16, 99%, 98.7 | 7 weeks pregnant by last menstrual period. Presents with vaginal bleeding for 5 hours, 2 pads used. Pending ultrasound. |  |  |
| P4 | P | 2 week M | Fever | 170, 72/50, 40, 98%, 101 | Labs and urine collected. IV line placed and received antibiotics. Pending lumbar punture. |  | Team should ask NICU to take patient and perform LP. |
| P5 | P | 19 yo M | Abdominal Pain | 106, 105/82, 26, 98%, 99.6 FS 340 mg/dL | ECG normal. VBG shows acidosis with K of 5.0. Pending labs. Receiving IV fluids. |  |  |
| P6 | P | 20 yo F | Vaginal Discharge | 82, 125/90, 18, 100%, 98.7 | Vaginal discharge for 3 days. Pending urinalysis. Treated for gonorrhea and chlamydia. |  |  |
| P7 | P | 2 yo M | Breathing Problem | 150, 90/60, 35, 94%, 98.6 | History of asthma. Presents with rhinorrhea, congestion, dyspnea for 2 days. Received 3 albuterol-ipratropium, orapred and magnesium IV. Currently receiving continuous albuterol. Wheezing on exam but overall improved (no accessory muscle use, much more comfortable). | Improves after continuous |  |
| P8 |  |  |  |  |  |  |  |
| P9 | DC | 9 yo M | Throat Pain |  |  |  |  |
| P10 | P | 5 yo F | Ear Pain | 110, 110/70, 22, 99%, 98.6 | Pain in left ear. Fever at home. Otitis media on exam. Pending a dose of amoxicillin and discharge. |  |  |
| PAs1 | P | 5 yo boy | Wheezing | 130, 110/72, 32, 93%, 98.6 | History of asthma. Presented with dyspnea for one day. Currently receiving albuterol-ipratropium and orapred. | Improves after duoneb and orapred. |  |
| PAs2 | P | 3 yo boy | Dyspnea | 130, 90/60, 32, 94%, 99.6 | History of asthma. Presents with dyspnea while playing outside. Currently receiving treatment of albuterol-ipratropium and appears comfortable. | Improves after 3 duonebs and orapred. |  |
| PAs3 | DC | 8 yo girl | Arm Pain | WNL |  |  |  |
| PAs4 | P | 12 yo boy | Ankle Pain | WNL | Riding bicycle when slammed foot down to stop self. Now unable to ambulate. Swelling, pain and obvious deformity of left ankle on exam. XR completed – demonstrating fracture and dislocation. Pending orthopedics consultation. | Reduced by ortho, casted and discharged. |  |
| T1 | P | 63 yo M | Assault | 108, 149/90, 18, 99%, 98.6 | Drinking etoh. Presented after assault with laceration to head, which was repaired; CT head and C spine negative, labs unremarkable, EtOH 280 (2 hours ago); Pending sobriety and tertiary exam. |  |  |
| FT1 | DC | 23 yo M | Sore Throat | WNL |  |  |  |
| FT2 | TBS | 22 yo F | Vaginal Discharge | WNL |  |  |  |
| FT3 | P | 27 yo M | Shoulder Pain | WNL | Pending XR. |  |  |
| FT4 | P | 61 yo M | High Blood Pressure | WNL | Pending labs. |  |  |
| FT5 | TBS | 39 yo F | Breast Problem | WNL |  |  |  |
| FT6 | DC | 35 yo F | Nasal Congestion | WNL |  |  |  |
| FT7 | TBS | 40 yo F | Vaginal Pain | WNL |  |  |  |
| FT8 | P | 46 yo M | Neck Pain | WNL | Pending analgesia administration. |  |  |
| FT9 | DC | 22 yo F | MVA | WNL |  |  |  |
| FT10 | P | 38 yo M | Back Pain | WNL | Pending analgesia administration. |  |  |
| FT11 | P | 41 yo M | Hand pain | WNL | Pending XR results. |  |  |
| FT12 | DC | 28 yo F | Pregnancy Test | WNL |  |  |  |
| FT13 |  |  |  |  |  |  |  |
| FT14 |  |  |  |  |  |  |  |
| FTC1 |  |  |  |  |  |  |  |
| FTC2 |  |  |  |  |  |  |  |
| FTC3 |  |  |  |  |  |  |  |
| FTC4 |  |  |  |  |  |  |  |
| FTC5 |  |  |  |  |  |  |  |
| FTC6 |  |  |  |  |  |  |  |
| WR |  | 28 yo M | Tooth Pain, Facial Swelling | 82, 111/60, 18, 100%, 98.2 |  |  |  |
| WR |  | 37 yo F | Joint Swelling | 85, 121/68, 20, 98%, 99.3 |  |  |  |
| WR |  | 50 yo M | Shoulder Pain | 95, 131/78, 18, 98%, 98.0 |  |  |  |
| WR |  | 36 yo F | Dizziness | 97, 102/57, 18, 100%, 98.1 |  |  |  |
| WR |  | 41 yo M | Knee Pain | 83, 149/76, 20, 100%, 98.5 |  |  |  |
| WR |  | 48 yo F | Hot Flashes | 81, 112/80, 20, 100%, 98.3 |  |  |  |
| WR |  | 23 yo F | Abscess | 86, 127/67, 18, 100%, 98.9 |  |  |  |
| WR |  | 64 yo F | Joint Swelling | 94, 153/91, 18, 100%, 98.1 |  |  |  |
| WR |  | 22 yo F | Exposure to STD | 92, 119/68, 18, 100%, 98 |  |  |  |
| WR |  | 52 yo F | Rash | 81, 121/68, 18, 97%, 99.3 |  |  |  |
| WR |  | 28 yo M | Pain, Back | 68, 111/68, 18, 99%, 98.1 |  |  |  |
| WR |  | 63 yo M | Eye Problem | 72, 159/91, 17, 96%, 98.4 |  |  |  |
| WR |  | 37 yo M | Leg Pain | 68, 129/82, 18, 97%, 98.3 |  |  |  |
| WR |  | 46 yo M | Breathing Problem | 95, 146/94, 20, 100%, 98.9, |  |  |  |
| WR |  | 58 yo M | Exposure to STD, Blood Sugar Problem | 94, 153/100, 18, 99%, 98.2, FS 597 mg/dL |  |  |  |
| WR |  | 36 yo M | Behavioral Problem | 92, 119/68, 18, 100%, 97.9 |  |  |  |

**Tabletop Exercise**

**Hospital Scene**

**Sign Out List for Participants**

**Key:**
Bed: A = Main Adult ED; AH = Main Hallway; AS = main asthma chair; C = crisis (psych ED); M = Midtrack area; P = pediatric ED, FT = fast track; FTC = Fast track chair; WR = waiting room
Status: A = admitted; P = pending disposition; DC = Discharged; P:obs = pending acceptance to observation unit; MC = medically cleared for psychiatric eval; TBS = To be seen;

CC = chief complaint; WNL = within normal limits; Vitals are presented in following order: Heart Rate, Blood Pressure, Respiratory Rate, Oxygen Saturation, Temperature (in Fahrenheit); FS = point of care glucose measurement; NC = nasal cannula, NRB = non-rebreather, BIPAP = bilevel positive airway pressure;

| **Bed** | **Status** | **Pt** | **CC** | **Vitals** | **Sign Out Notes** |
| --- | --- | --- | --- | --- | --- |
| A1 | A | 63 yo M | Dyspnea | 110, 120/82, 18, 92%, 98.8, FS 98 | CHF exacerbation. Receiving dialysis in emergency department. |
| A2 | TBS | 76 yo F | Chest Pain | 98, 156/87, 18, 100%, 98.7 | Complaining of chest pain for 2 hours. History of MI. |
| A3 | A | 73 yo F | Cough, Fever | 93, 164/97, 22, 93% on NC, 100.3 | Rule out tuberculosis. |
| A4L | MC | 34 yo F | Behavioral Problem | WNL | Suicidal ideations |
| A4R | MC | 38 yo M | Alcohol Problem | WNL | Was intoxicated, now sober; Complaining of suicidal ideations. |
| A5 | MC | 49 yo M | Behavioral Problem | WNL | Aggressive with family at home, history of schizophrenia. |
| A6 | DC | 30 M | Abdominal Pain | WNL | Presented with nausea/vomiting/abdominal pain; CT abdomen/pelvis negative; Tolerated PO. |
| A7 | P | 54 yo M | Chest Pain | 88, 172/95, 18, 100%, 98.7 | Pending 2nd troponin (to be drawn in 1 hour) and ECG; HEART score of 2. Everything else negative. |
| A8 | P | 38 yo M | Asthma | 112, 139/94, 24, 98% on BIPAP, 99.3 | Currently on BIPAP; Wants to leave against medical advice. |
| A9 | A | 82 yo M | Cough | 110, 158/87, 26, 91% on NC, 100.5 | Admitted to step down unit for 2 days. |
| A10 | P | 63 yo M | Seizures | 90, 105/86, 12, 100% on ventilator, T99.4 | Presented with status epilepticus for 20 minutes, intubated for airway protection; on propofol and fentanyl drips; Pending labs and head CT |
| A11 | P | 33 yo F | Fever, Chills  Sore throat | 103, 129/84, 14, 100%, 101.4 | Exam: No muffled voice, difficulty breathing; Tolerating secretions; Large edema of the right peritonsillar space, erythematous, no plaques on tonsils.  Labs unremarkable  Peritonsillar abscess on bedside ultrasound. Pending drainage. |
| A12 | A | 67 yo F | Abdominal pain | 102, 148/94, 18, 99%, 100.6 | Admitted to surgery for acute cholecystitis. Receiving antibiotics. |
| A13 |  |  |  |  |  |
| A14 | P | 58 yo M | Dyspnea | 127, 188/105, 30, 94% on BIPAP, 99.1 | Presented 10 minutes ago in respiratory distress with diffuse rales and hypertensive to 200/115. Immediately placed on BIPAP and started nitroglycerin drip. Pt appears mildly more comfortable at this time. ECG: T wave inversions; BIPAP settings: 10/5 60% RR12 |
| A15 | A | 38 yo M | Abdominal Pain | 99, 110/80, 26, 98%, 100.2,  FS 540 mg/dL | Admitted to ICU for severe DKA. Receiving insulin drip with bicarb infusion. |
| A16 | DC | 56 yo M | Edema | WNL | Diagnosed with cellulitis, plan to discharged home with oral antibiotics. Labs/imaging unremarkable. |
| A17 | P | 86 yo M | AMS | 92, 156/97, 18, 98%, 99.3 | History of dementia and frequent falls; Brought in by family for worsening bizarre behavior, increasing forgetfulness.  History of A fib on warfarin. |
| A18 | P | 56 yo M | Blood Sugar Problem | 102, 148/92, 18, 99%, 98.6 FS 430 mg/dL | Noticed FS at home was high but otherwise asymptomatic. ECG normal, VBG normal, pending rest of labs. Receiving intravenous fluids. |
| A19 | P | 63 yo M | Chest Pain | 78, 160/90, 18, 99%, 98.5  FS 103 mg/dL | History of diabetes, CAD, HTN who presented with chest pain for one day. ECG and CXR are normal, pending labs. |
| A20 | P | 48 yo F | Drug Problem | 107, 164/96, 10, 93% on NC, 99.5,  FS 90 md/dL | Brought in by EMS after opioid overdose. Received 0.4 mg IV naloxone in the field. Now breathing and drowsy. |
| A21 | A | 82 yo F | Breathing Problem | 110, 150/80, 20, 94%, 101.2 | Presented with shortness of breath and weakness. Recently treated for pneumonia in hospital. Now also complaining of multiple episodes of diarrhea; Admitted for rule out C diff. |
| AH1 | TBS | 38 yo M | Back Pain | 68, 130/80, 18, 98%, 98.8 | Back pain since heavy lifting yesterday |
| AH2 | P | 48 yo F | Alcohol Problem | WNL | One of our regular patients admits to ETOH use today. No signs of trauma. |
| AH3 | P | 47 yo F | Opioid Overdose | 70, 148/96, 12, 99%, 98.7  FS 109 mg/dL | Received naloxone in field by EMS. Sitting in bed awake and alert, denies using heroin or other drugs, wants to leave. |
| AH4 | MC | 29 yo M | Behavioral Problem | WNL | Complaining of hallucinations. |
| AH5 | P | 23 yo F | Vaginal Bleed | 82, 102/80, 16, 99, 98.6 | 8 weeks pregnant with spotting for one day. Confirmed intrauterine pregnancy on ultrasound. Pending labs. |
| AH6 | P | 61 yo F | Suicidal Ideations | WNL | History of suicide attempt in past. Complains of suicidal ideations with plan. |
| AH7 |  |  |  |  |  |
| AH8 | P: Obs | 29 yo F | Vomiting | WNL | Presented with vomiting and right lower quadrant pain for one day. Labs and CT negative. Pending transfer to observation for hydration and PO challenge. |
| AH9 | TBS | 33 yo M | Rash | 90, 110/80, 18, 98%, 98.9 | Triage note: Rash for 2 days |
| AH 10 |  |  |  |  |  |
| AH 11 |  |  |  |  |  |
| AH 12 |  |  |  |  |  |
| AS1 | P | 55 yo M | Breathing Problem | 110, 120/90, 22, 92%, 98.7 | One of our regular patients with wheezing on exam. |
| AS2 | P | 58 yo F | Breathing Problem | 105, 150/98, 18, 93%, 98.9 | One of our regular patients with wheezing on exam. |
| C1 | MC | 38 yo F | Behavioral Problem | WNL | Suicidal ideations |
| C2 | MC | 49 yo M | Behavioral Problem | WNL | Brought in by police for aggressive behavior in street. Received 5 mg haldol and 5 mg versed IM. |
| C3 | MC | 29 yo F | Behavioral Problem | WNL | Hallucinations |
| C4 | MC | 37 yo M | Behavioral Problem | WNL | Suicidal ideations |
| C5 |  |  |  |  |  |
| C6 | MC | 53 yo F | Behavioral Problem | WNL | Suicidal ideations |
| M1 | TBS | 47 yo F | Leg Swelling |  |  |
| M2 | TBS | 24 yo M | Leg Pain |  | Obvious deformity on exam. |
| M3 | TBS | 55 yo M | Back Pain |  |  |
| M4 | TBS | 32 yo F | Abdominal Pain |  |  |
| M5 |  |  |  |  |  |
| M6 |  |  |  |  |  |
| P1 |  |  |  |  |  |
| P2 | DC | 7 yo M | Fall | WNL |  |
| P3 | P | 19 yo F | Vaginal Bleeding | 90, 105/80, 16, 99%, 98.7 | 7 weeks pregnant by last menstrual period. Presents with vaginal bleeding for 5 hours, 2 pads used. Pending ultrasound. |
| P4 | P | 2 week M | Fever | 170, 72/50, 40, 98%, 101 | Labs and urine collected. IV line placed and received antibiotics. Pending lumbar punture. |
| P5 | P | 19 yo M | Abdominal Pain | 106, 105/82, 26, 98%, 99.6 FS 340 mg/dL | ECG normal. VBG shows acidosis with K of 5.0. Pending labs. Receiving IV fluids. |
| P6 | P | 20 yo F | Vaginal Discharge | 82, 125/90, 18, 100%, 98.7 | Vaginal discharge for 3 days. Pending urinalysis. Treated for gonorrhea and chlamydia. |
| P7 | P | 2 yo M | Breathing Problem | 150, 90/60, 35, 94%, 98.6 | History of asthma. Presents with rhinorrhea, congestion, dyspnea for 2 days. Received 3 albuterol-ipratropium, orapred and magnesium IV. Currently receiving continuous albuterol. Wheezing on exam but overall improved (no accessory muscle use, much more comfortable). |
| P8 |  |  |  |  |  |
| P9 | DC | 9 yo M | Throat Pain |  |  |
| P10 | P | 5 yo F | Ear Pain | 110, 110/70, 22, 99%, 98.6 | Pain in left ear. Fever at home. Otitis media on exam. Pending a dose of amoxicillin and discharge. |
| PAs1 | P | 5 yo boy | Wheezing | 130, 110/72, 32, 93%, 98.6 | History of asthma. Presented with dyspnea for one day. Currently receiving albuterol-ipratropium and orapred. |
| PAs2 | P | 3 yo boy | Dyspnea | 130, 90/60, 32, 94%, 99.6 | History of asthma. Presents with dyspnea while playing outside. Currently receiving treatment of albuterol-ipratropium and appears comfortable. |
| PAs3 | DC | 8 yo girl | Arm Pain | WNL |  |
| PAs4 | P | 12 yo boy | Ankle Pain | WNL | Riding bicycle when slammed foot down to stop self. Now unable to ambulate. Swelling, pain and obvious deformity of left ankle on exam. XR completed – demonstrating fracture and dislocation. Pending orthopedics consultation. |
| T1 | P | 63 yo M | Assault | 108, 149/90, 18, 99%, 98.6 | Drinking etoh. Presented after assault with laceration to head, which was repaired; CT head and C spine negative, labs unremarkable, EtOH 280 (2 hours ago); Pending sobriety and tertiary exam. |
| FT1 | DC | 23 yo M | Sore Throat | WNL |  |
| FT2 | TBS | 22 yo F | Vaginal Discharge | WNL |  |
| FT3 | P | 27 yo M | Shoulder Pain | WNL | Pending XR. |
| FT4 | P | 61 yo M | High Blood Pressure | WNL | Pending labs. |
| FT5 | TBS | 39 yo F | Breast Problem | WNL |  |
| FT6 | DC | 35 yo F | Nasal Congestion | WNL |  |
| FT7 | TBS | 40 yo F | Vaginal Pain | WNL |  |
| FT8 | P | 46 yo M | Neck Pain | WNL | Pending analgesia administration. |
| FT9 | DC | 22 yo F | MVA | WNL |  |
| FT10 | P | 38 yo M | Back Pain | WNL | Pending analgesia administration. |
| FT11 | P | 41 yo M | Hand pain | WNL | Pending XR results. |
| FT12 | DC | 28 yo F | Pregnancy Test | WNL |  |
| FT13 |  |  |  |  |  |
| FT14 |  |  |  |  |  |
| FTC1 |  |  |  |  |  |
| FTC2 |  |  |  |  |  |
| FTC3 |  |  |  |  |  |
| FTC4 |  |  |  |  |  |
| FTC5 |  |  |  |  |  |
| FTC6 |  |  |  |  |  |
| WR |  | 28 yo M | Tooth Pain, Facial Swelling | 82, 111/60, 18, 100%, 98.2 |  |
| WR |  | 37 yo F | Joint Swelling | 85, 121/68, 20, 98%, 99.3 |  |
| WR |  | 50 yo M | Shoulder Pain | 95, 131/78, 18, 98%, 98.0 |  |
| WR |  | 36 yo F | Dizziness | 97, 102/57, 18, 100%, 98.1 |  |
| WR |  | 41 yo M | Knee Pain | 83, 149/76, 20, 100%, 98.5 |  |
| WR |  | 48 yo F | Hot Flashes | 81, 112/80, 20, 100%, 98.3 |  |
| WR |  | 23 yo F | Abscess | 86, 127/67, 18, 100%, 98.9 |  |
| WR |  | 64 yo F | Joint Swelling | 94, 153/91, 18, 100%, 98.1 |  |
| WR |  | 22 yo F | Exposure to STD | 92, 119/68, 18, 100%, 98 |  |
| WR |  | 52 yo F | Rash | 81, 121/68, 18, 97%, 99.3 |  |
| WR |  | 28 yo M | Pain, Back | 68, 111/68, 18, 99%, 98.1 |  |
| WR |  | 63 yo M | Eye Problem | 72, 159/91, 17, 96%, 98.4 |  |
| WR |  | 37 yo M | Leg Pain | 68, 129/82, 18, 97%, 98.3 |  |
| WR |  | 46 yo M | Breathing Problem | 95, 146/94, 20, 100%, 98.9, |  |
| WR |  | 58 yo M | Exposure to STD, Blood Sugar Problem | 94, 153/100, 18, 99%, 98.2, FS 597 mg/dL |  |
| WR |  | 36 yo M | Behavioral Problem | 92, 119/68, 18, 100%, 97.9 |  |

**Tabletop Exercise – Patients to be Triaged in ED**

**Hospital Scene**

**Victims from Mass Casualty Incident**

Instructions:

Each patient indicated by a number. Cut out each patient description and past on index card. Do not include triage color on index card. Be sure to include number. That will allow you to refer to this answer key that participants are completing proper tagging. Some patients have changes on reassessment. Cut these descriptions and place on separate index card. Numbers will allow you to place the correct reassessment description on top of the right patient. Do this when indicated by the facilitator guide.

| **Patient, CC/Profile** | **Initial Tag Color** | **Reassessment** | **Reassessment Tag Color** | **Notes for Facilitator/Dx** |
| --- | --- | --- | --- | --- |
| 1) 30 y/o M burns to arms and torso (appears 2nd degree). Able to describe what happened.  HR 136, RR 29, O2: 96%, BP: 168/110 | Yellow |  | N/A |  |
| 2) 35 y/o F with dyspnea and history of asthma. Walked into ED, but states she lives in building where fire occurred.  HR 101, RR 32, O2: 94%, BP 146/87 | Green | 2) Patient even more tachypneic with desaturation to 80s. Not answering questions appropriately. | Red | first to present--decompensating respiratory status, becomes altered--inhalational injury |
| 3) 9 y/o M object fell on him and mother. Obvious arm deformity. Able to walk. | Green |  | N/A | broken arm |
| 4) 36 y/o F hit in back of head by falling object. Brief loss of consciousness. Complaining now of headache. One episode of vomiting. Palpable radial pulse.  Vitals: HR 102, RR 20,  O2 98%, BP 150/90 | Yellow | 4) On recheck, patient found to be somnolent. Difficult to arouse and does not follow commands. CT head showing epidural hematoma | Red | head bleed---if no CT quick enough will get AMS |
| **Patient, CC/Profile** | **Initial Tag Color** | **Reassessment** | **Reassessment Tag Color** | **Notes for Facilitator/Dx** |
| 5) 72 y/o F who was trapped in apartment for 45mins after ceiling collapsed on her. Complaining to you of chest pain and dyspnea with shortened and externally rotated left lower extremity. Good capillary refill.  Vitals: HR 110, RR 25, O2 94%, BP 103/80 | Yellow |  | N/A |  |
| 6) Young male with extensive burns to face and unresponsive. EMS unable to intubate. | Red |  | N/A |  |
| 7) 32 y/o firefighter presents after face mask knocked with coughing. Able to walk. | Green |  | N/A |  |
| 8) 7month old, evacuated with mother, crying. Palpable pulses. Alert.  Vitals: HR 140, RR 35, O2 sat 98%, BP 72/54 | Green |  | N/A |  |
| 9) Middle aged female discovered to be spontaneously breathing. She is making incomprehensible sounds and does not open her eyes. She withdraws to painful stimuli. You note full thickness burns throughout bilateral lower and upper extremities (anterior and posterior), entire anterior trunk, genitals and lower posterior trunk. | Red |  | N/A |  |
| **Patient, CC/Profile** | **Initial Tag Color** | **Reassessment** | **Reassessment Tag Color** | **Notes for Facilitator/Dx** |
| 10) Young adult male with injuries to his left arm and right leg. There is active bleeding from both sites. The lower extremity has a partial amputation. Partial and full thickness burns to the anterior lower extremities and trunk. He does not answer questions or follow commands, but is breathing spontaneously at a rate of 24. Palpable radial pulses are present. | Red |  | N/A |  |
| 11) Young adult male found pinned under rubble. Mangled and partially amputated lower extremity. His eyes are open and he is confused. There is active bleeding noted from the amputation site. Radial pulses are not palpable. | Red |  | N/A |  |
| 12) Young adult female with full thickness burns to the anterior left upper extremity and partial thickness burns to the anterior left lower extremity. She complains of left abdominal pain and back pain. She is able to follow commands and has palpable radial pulses. She is not able to ambulate.  Vitals: HR 108, RR 22, O2 100%, BP 98/58 | Yellow | 12) She is now diaphoretic and confused but repeatedly moaning in pain. She is moving her extremities spontaneously and opens her eyes to voice. Vitals: HR 132, RR 32, O2 92%, BP 82/60 | Red |  |
| **Patient, CC/Profile** | **Initial Tag Color** | **Reassessment** | **Reassessment Tag Color** | **Notes for Facilitator/Dx** |
| 13) Young adult male who is walking but holding his arm with an obvious deformity. | Green |  | N/A |  |
| 14) Middle aged male walks in with burns to bilateral hands after touching hot door while attempting to escape. | Green |  | N/A |  |
| 15) 71 y/o M was struck in the abdomen while escaping. He complains of abdominal pain and obeys commands. Capillary refill is 1 second.  Vitals: HR 110, RR 16, O2 96%, BP 110/85, | Yellow | 15) Repeat Vitals: HR 137, BP 87/56, RR 32, 94%, FAST+ | Red | Needs blood transfusion for bleeding within abdomen. |
| 16) 15 y/o girl pinned on stairs and stepped on by multiple people in rush to get out of building. Complaining of rib pain. Able to ambulate. | Green | 16) CXR resulted: right sided pneumothorax. Patient complaining of increased shortness of breath.  Vitals: HR 120, RR 20, O2 89%, BP 89/60 | Yellow | CXR showing rib fx and pneumothorax |
| 17) 64 y/o slipped on ice outside, wrist pain. Able to ambulate. | Green |  | N/A |  |
| 18) 65 y/o female found unconscious outside away from building. Unknown how long she was there. Now awake and alert, but shivering. She says she has a history of fainting spells. Unable to ambulate. Palpable radial pulse.  Vitals: HR 120, RR 20, O2 96%, BP 134/86, T 94. 3, | Yellow |  | N/A | hypothermic |
| **Patient, CC/Profile** | **Initial Tag Color** | **Reassessment** | **Reassessment Tag Color** | **Notes for Facilitator/Dx** |
| 19) 8 y/o boy arm pain. Able to ambulate. | Green |  | N/A |  |
| 20) 17 y/o F at 41 weeks gestation startled awake. Able to ambulate. Reporting contractions. | Green |  | N/A |  |
| 21) 3 y/o boy who was sleeping at time of fire, now screaming and crying for parents. Palpable pulses.  Vitals: HR 120, RR 28, O2 100%, BP 85/60 | Green |  | N/A |  |
| 22) 62 y/o male found asleep in floor below fire. Ambulates with walker and complains of foot pain and lacerations after walking through glass without shoes on. | Green |  | N/A |  |
| 23) 26 y/o male ran out of building but slipped on icy steps. Now with back and ankle pain, hopping on 1 foot. | Green |  | N/A |  |
| 24) 23 y/o female with history of anxiety presents with shakiness, crying and hyperventilating. Able to ambulate. | Green |  | N/A |  |

**Patients Presenting to ED NOT via EMS (Walk-Ins)**

| **Patient Description** | **Triage Color** | **Notes for Facilitator** |
| --- | --- | --- |
| 68 yo F Weakness  BP 168/90, HR 90, RR 19, O2 98% RA, T 98.6  Was eating lunch with family 1 hour ago when had sudden onset weakness of the L arm and collapsed, now moaning, opens eyes to pain, withdraws to pain on R, no movement on L | N/A | Stroke patient  Group should recognize that patient needs to be intubated and receive tPA |
| 30 yo F Headache  BP 120/80, HR 90, RR 22, O2 94% RA, T 98.8  Was on scene during the fire, escaped before building collapsed, immediately presented to ED; Complaining of lightheadedness and headache.  First degree burns on legs. | Green |  |
| 18 yo M Arm pain  BP 150/80, HR 99, RR 26, O2 99% RA, T 99.3  At scene of building collapse. Walked away from first responders. Presents with obvious arm deformity. | Green |  |
| 47 yo F Abdominal pain  BP 88/60, HR110, RR20, O2 99% RA, T 98.5  Escaped from rubble of building collapse. A friend drove her in car. Complaining of diffuse abdominal pain, bruising and abrasions, first degree burns on arms. Unable to walk. Palpable distal pulses. | Yellow |  |
| 18 yo F Headache  BP 110/78, HR 80, RR 24, O2 94% RA, T 98.7  Was in the fire initially but escaped before building collapsed. Drove self to the ED. Complaining of headache. | Green |  |

**Tabletop Exercise – Hospital Resources List**

Instructions: Cut out each piece of equipment. Place on map of emergency department as available to participants for “treatment” of patients.

| **Endotracheal Tube** | **Portable Monitor** | **Ventilator** | **Tourniquet** |
| --- | --- | --- | --- |
| **Endotracheal Tube** | **Portable Monitor** | **Ventilator** | **Tourniquet** |
| **Endotracheal Tube** | **Portable Monitor** | **Ventilator** | **Tourniquet** |
| **Endotracheal Tube** | **Portable Monitor** | **Ventilator** | **Tourniquet** |
| **Endotracheal Tube** | **Portable Monitor** | **Ventilator** | **Tourniquet** |
| **Endotracheal Tube** | **Chest Tube** | **Ventilator** | **Tourniquet** |
| **Endotracheal Tube** | **Chest Tube** |  | **Tourniquet** |
| **Endotracheal Tube** | **Chest Tube** |  | **Tourniquet** |
| **Endotracheal Tube** | **Chest Tube** |  | **Tourniquet** |
| **Endotracheal Tube** | **Chest Tube** |  |  |

**Tabletop Exercise – Participant Instructions
Hospital Scene: Sheet 1**

Scenario:

It is Friday, February 24^th^ at 9:45PM. EMS informs you that there is an apartment fire nearby in a large high-rise building, and you should anticipate a large number of patients. You do not know how many could be coming at this point. The scene is still being processed, and the number of patients and their acuity is unknown. You must quickly prepare for a large influx of patients from the scene.

Staff Currently Present in ED:

| **Staff Qualification** | **Number Present** | **Staff Qualification** | **Number present** |
| --- | --- | --- | --- |
| EM Attendings: | 3 Adult attendings | Nurse Practitioners | 2 |
|  | 1 Ped attending | Nurses | 23 |
| Trauma Surgeons: | 1^st^ - in Hospital | Nurse Techs | 7 |
|  | 2^nd^ - Home Call |  |  |
| EM Residents: |  |  |  |
| EM4 | 1 | 4^th^-year Medical Students | 1 |
| EM3 | 2 | IM Residents (PGY-2) | 2 |
| EM2 | 2 |  |  |
| EM1 | 3 |  |  |

You will find maps of the emergency department in front of you with the current patients in each section of the department.

**Tasks**:

1. Prepare for arrival of patients from the scene.

1. As the emergency department, you are part of the operations section. Assign roles amongst your staff such as who will be the attending physician, resident physicians, charge nurse, etc. What specific jobs will each person be doing when patients arrive?
2. Where will EMS bring patients?
3. Where are you going to put patients?
4. How are you going to decide who will care for who?
5. How will you continue to care for the patients already in the emergency department?

**Tabletop Exercise – Participant Instructions**

**Hospital Scene: Sheet 2**

Patients begin arriving to the emergency department. You are told that there was a building collapse and patients are starting to be pulled from the rubble. Simultaneously, many physicians and nurses begin to arrive to the emergency department from home and begin to clog the main ED and trauma bay. Also, members of the public arrive looking for family members they are worried were in the collapse.

Does your current plan work for the incoming patients? What adjustments can be made?

What are you going to do with the extra staff? How will you verify their credentials?

How are you going to rapidly register the large volume of patients?

How will you handle the family members?
