# Supplementary material for: Disaster Preparedness Training for Emergency Medicine Residents Using a Tabletop Exercise
Source: MedEdPORTAL. 2021 Mar 12;17:11119. doi: 10.15766/mep_2374-8265.11119 (PMC7970644; doi:10.15766/mep_2374-8265.11119)
Supplement: Supplementary file 1 — Exercise Lecture.pptxDisaster Scene Packet.docxHospital Scene Packet.docxPre-Exercise Survey.docxPostexercise Survey.docx [file mep_2374-8265.11119-s001.zip › D. Pre-Exercise Survey.docx]

**Tabletop Exercise**

**Pre-Exercise Survey**

1. What year of EM residency is this for you?
   1. 1
   2. 2
   3. 3
   4. 4
2. There is an explosion at a local mall. You are first to arrive on scene and are told there are hundreds of people potentially injured. You start triaging patients using the Simple Triage and Rapid Treatment (START) technique. The first patient is a young woman lying on the ground. She has bilateral palpable radial pulses, heart rate 125, respiratory rate 26, mild facial trauma, and an obvious left lower extremity deformity. She is alert but does not follow commands. Which color triage tag is appropriate for this patient?
   1. Black
   2. Green
   3. Red
   4. Yellow
3. A patient arrives in the Emergency Department from a factory explosion. The patient has a piece of machinery fragment stuck into his left thigh. Bleeding has been controlled by EMS. The patient’s left leg has normal pulses, strength, and sensation. This injury is an example of what type of blast injury?
   1. Primary Blast Injury
   2. Secondary Blast Injury
   3. Tertiary Blast Injury
   4. Quaternary Blast Injury
4. There has been a large train derailment, with hundreds of injured passengers. You are assigned to triage patients on scene according to the Simple Triage and Rapid Treatment (START) technique. The first patient is an adult who is unconscious with significant head and facial trauma and no spontaneous respirations. What is the most appropriate next step?
   1. Assign the patient a black tag
   2. Intubate the patient
   3. Reposition the airway
   4. Assign the patient a red tag
   5. Oxygenate the patient with a bag valve mask
5. You are on scene at a highway crash involving two large passenger busses with dozens of injured people. The first patient you encounter has spontaneous respirations at 24 per minute and a large left calf laceration. His left leg has present but diminished distal pulse with delayed capillary refill. Based on the START protocol, which color tag should be assigned to this patient?
   1. Green
   2. Yellow
   3. Red
   4. Black
6. Which of the following elements of a hospital’s disaster response plan is responsible for securing resources and supplies in support of the incident?
   1. Logistics
   2. Finance and administration
   3. Operations
   4. Planning
7. EMS arrives on the scene of a motor vehicle collision with a tree. The driver is the only the patient. There is a local hospital one mile from the site, and a trauma center 25 miles away. Which of the following findings should prompt transport to the trauma center instead of the local hospital?
   1. The patient has an isolated forearm deformity.
   2. The patient is 16 weeks pregnant.
   3. The patient takes warfarin daily.
   4. The patient's initial blood pressure is 100/80
8. Which level trauma center is fully equipped and staffed by emergency physicians but is without 24-hour immediate coverage of specialty and surgical services?
   1. Level I
   2. Level II
   3. Level III
   4. Level IV
9. Which is the minimum emergency medical services provider certification level is needed in order to start an IV line?
   1. EMT-Basic
   2. EMT-Intermediate
   3. EMT-Paramedic
   4. First Responder

Use the scenario below to answer questions 10-11 on the 5-point Likert scale.

You are the only physician at a small community hospital in the middle of the night.  There is a local train crash and derailment.  There are expected to be at least 70 victims.  Your hospital is not a trauma center, but the nearest trauma center is a 3-hour drive and many of the victims will be coming to your hospital to be stabilized.  Rank your level of agreement with the following statements.

1. I am confident in my ability to hand an incident such as this.
   1. Strongly disagree
   2. Disagree
   3. Neither agree nor disagree
   4. Agree
   5. Strongly Agree
2. The subject of disaster medicine is important to emergency medicine
   1. Strongly disagree
   2. Disagree
   3. Neither agree no disagree
   4. Agree
   5. Strongly Agree
3. Do you feel that your education and training in residency has prepared you adequately to handle this type of incident?
   1. I have received no formal education or training on this but feel prepared
   2. I have received no formal education or training and do not feel at all prepared
   3. I have received some formal education or training but could use more.
   4. I have received formal education or training and am adequately prepared.

**Tabletop Exercise**

**Pre-Exercise Survey Answer Key**

1. C. Red
2. B. Secondary Blast Injury
3. C. Reposition the patient’s airway
4. C. Red
5. A. Logistics
6. C. Patient takes warfarin daily
7. C. Level III
8. C. EMT-Paramedic
9. B. 31 year old male with vomiting approximately 20 minutes after the explosion
