# Supplementary material for: Disaster Preparedness Training for Emergency Medicine Residents Using a Tabletop Exercise
Source: MedEdPORTAL. 2021 Mar 12;17:11119. doi: 10.15766/mep_2374-8265.11119 (PMC7970644; doi:10.15766/mep_2374-8265.11119)
Supplement: Supplementary file 1 — Exercise Lecture.pptxDisaster Scene Packet.docxHospital Scene Packet.docxPre-Exercise Survey.docxPostexercise Survey.docx [file mep_2374-8265.11119-s001.zip › E. Postexercise Survey.docx]

**Tabletop Exercise**

**Post-Exercise Survey**

1. An explosion at a nearby nuclear power plant releases a significant amount of gamma radiation into the nearby community. Multiple patients present to your Emergency Department for possible radiation poisoning. Which of the follow patients is most critical?
   1. 16-year-old girl with vomiting, fatigue, and lethargy approximately 3 hours after the explosion
   2. 31-year-old man with vomiting approximately 20 minutes after the explosion
   3. 45-year-old woman with headache approximately 45 minutes after the explosion
   4. 6-year-old man with fatigue approximately 2 hours after the explosion
   5. 64-year-old woman who experiences cough and mild shortness of breath approximately 1 hour after the explosion.
2. Which of the following patients must be brought to the nearest trauma center?
   1. 54-year-old woman restrained backseat passenger without LOC and without any complaints at this time in a head-on collision of 2 vehicles going 30 mph. The driver of the patient’s vehicle was dead on scene.
   2. 26-year-old asymptomatic pregnant woman at 18 weeks gestation, restrained front seat passenger in a car rear-ended while stopped at a traffic light.
   3. A 30-year-old man restrained passenger in an MVC without airbag deployment or LOC, found to have an isolated R wrist deformity.
   4. A 42-year-old woman driver of a single vehicle collision with a tree with an initial BP of 100/80.
3. According to the START Adult triage algorithm, what color would you tag the following patient?

32 yo F with obvious arm deformity, who is able to ambulate, speaking in full sentences, following commands and breathing at a respiratory rate of 28 with intact radial pulses.

- 1. Green
  2. Yellow
  3. Red
  4. Black

1. What is the role of the staging area in the incident command system?
   1. where personnel gather to be organized and receive their individual assignments and tasks
   2. where the chiefs of each section (operations, planning, logistics and finance) gather to coordinate the response
   3. where patients are gathered to prepare transport to nearby hospital
   4. where the incident commander is located
2. What is one difference between a level I and a level II trauma center?
   1. Level I trauma centers see more serious injured patients
   2. Level II trauma centers do not have 24 hour coverage by surgical services
   3. Level II trauma centers have 24 hour coverage by surgical services but do not treat children
   4. Level I trauma center must operate an organized research and teaching effort to contribute to new innovations in trauma care
3. A patient presents to you who was the victim of an explosion at a nearby train station.  Part of a wall fell as a result of the explosion and crushed his arm.  This is an example of what type of blast injury?
   1. Primary
   2. Secondary
   3. Tertiary
   4. Quaternary
4. You are on scene of a plane crash when you find a victim who does not appear to be breathing.  What do you do next?
   1. Reposition the airway
   2. Mark the patient as a black tag. There are many other potential critical patients.
   3. Immediately call for help and attempt to intubate the patient.
   4. Check for a pulse

Use the scenario below to answer the next three questions on a 5-point Likert scale.

You are the only physician at a small community hospital in the middle of the night.  There is a local train crash and derailment.  There are expected to be at least 70 victims.  Your hospital is not a trauma center, but the nearest trauma center is a 3-hour drive and many of the victims will be coming to your hospital to be stabilized.

1. I am confident in my ability to handle an incident such as this.
   1. Strongly disagree
   2. Disagree
   3. Neither agree nor disagree
   4. Agree
   5. Strongly agree
2. The subject of disaster medicine is important to emergency medicine.
   1. Strongly disagree
   2. Disagree
   3. Neither agree nor disagree
   4. Agree
   5. Strongly agree
3. I believe that a tabletop exercise is an effective education tool for disaster preparedness and training.
   1. Strongly disagree
   2. Disagree
   3. Neither agree nor disagree
   4. Agree
   5. Strongly agree

**Tabletop Exercise**

**Pre-Exercise Survey Answer Key**

1. b. 31-year-old man with vomiting approximately 20 minutes after the explosion
2. a. 54-year-old woman restrained backseat passenger without LOC and without any complaints at this time in a head-on collision of 2 vehicles going 30 mph. The driver of the patient’s vehicle was dead on scene.
3. a. Green
4. a. where personnel gather to be organized and receive their individual assignments and tasks
5. d. Level I trauma center must operate an organized research and teaching effort to contribute to new innovations in trauma care
6. c. Tertiary
7. a. Reposition the airway
